# Mechanical couplings of protein backbone and side chains exhibit scale-free network properties and specific hotspots for function

**DOI:** 10.1016/j.csbj.2021.09.004

**Published:** 2021-09-08

**Authors:** Nixon Raj, Timothy Click, Haw Yang, Jhih-Wei Chu

**Affiliations:** a Institute of Bioinformatics and Systems Biology, National Yang Ming Chiao Tung University, 75 Bo-Ai Street, Hsinchu 30010, Taiwan, ROC; b Department of Biological Science and Technology, National Yang Ming Chiao Tung University, 75 Bo-Ai Street, Hsinchu 30010, Taiwan, ROC; c Institute of Molecular Medicine and Bioengineering, National Yang Ming Chiao Tung University, 75 Bo-Ai Street, Hsinchu 30010, Taiwan, ROC; d Center for Intelligent Drug Systems and Smart Bio-devices (IDS^2^B), National Yang Ming Chiao Tung University, 75 Bo-Ai Street, Hsinchu 30010, Taiwan, ROC; e Department of Chemistry, Princeton University, Princeton, NJ 08544, USA

**Keywords:** Structure-mechanics statistical learning, Network theory, Rigidity graph, Protein dynamics, All-atom molecular dynamics simulation, Scale-free, Serine protease, PDZ3

## Abstract

A backbone-side-chain elastic network model (bsENM) is devised in this contribution to decipher the network of molecular interactions during protein dynamics. The chemical details in 5 μs all-atom molecular dynamics (MD) simulation are mapped onto the bsENM spring constants by self-consistent iterations. The elastic parameters obtained by this structure-mechanics statistical learning are then used to construct inter-residue rigidity graphs for the chemical components in protein amino acids. A key discovery is that the mechanical coupling strengths of both backbone and side chains exhibit heavy-tailed distributions and scale-free network properties. In both rat trypsin and PDZ3 proteins, the statistically prominent modes of rigidity graphs uncover the sequence-specific coupling patterns and mechanical hotspots. Based on the contributions to graphical modes, our residue rigidity scores in backbone and side chains are found to be very useful metrics for the biological significance. Most functional sites have high residue rigidity scores in side chains while the biologically important glycines are generally next to mechanical hotspots. Furthermore, prominent modes in the rigidity graphs involving side chains oftentimes coincide with the co-evolution patterns due to evolutionary restraints. The bsENM specifically devised to resolve the protein chemical character thus provides useful means for extracting functional information from all-atom MD.

## Highlights

• Statistical learning from protein dynamics unravels rigidities in interaction network.
• Backbone and side-chain mechanical couplings exhibit scale-free network properties.
• Graphical depiction of network rigidities captures sequence co-evolution patterns.
• Functional sites at secondary structure peripheries are mechanical hotspots.
• Our rigidity scores are compelling metrics for residue biological significance.

## Graphical abstract

**Figure:**
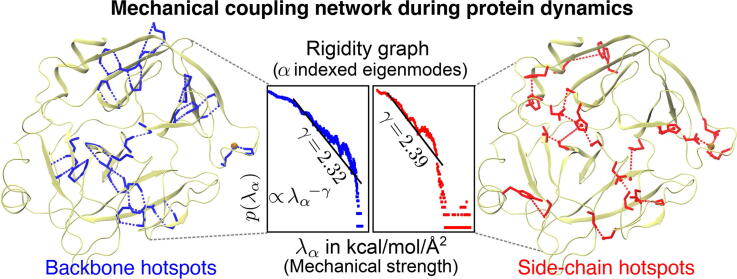

## 1 Introduction

Proteins exhibit remarkable properties such as thermal stability, specific molecular binding, and catalytic activities. These functionally important features are sensitive to mutation and can trace their origin to both the polypeptide backbone that frames the structure and the side chains that define the chemical specificity [1], [2], [3]. Deciphering the contributions of these two components to functional properties, though, remains a fundamental challenge [4], [5], [6]. The folded topology was recognized to host a variety of sequences in structural families [7], [8] and has inspired artificial protein engineering and design [9], [10], [11]. Constructing potential energy function with the structural network, such as the elastic network model (ENM) [12], [13], [14] and the Gō model [15], [16], [17], is very useful in studying functional motions [12], [13], [14], protein folding [15], [16], [17], and allosteric wiring [18], [19], [20]. In addition to the Hamiltonian-based methods, graphical analysis [21], [22], [23] is frequently applied to analyze the protein structural network. If the distance between a residue pair is within a cutoff, their edge in the adjacency matrix A is typically set to one, and the diagonal degree matrix D records the residue contact numbers. This topology-based approach corresponds to using a universal spring constant in ENM. The Laplacian matrix (L=D-A) [24], [25] was found to offer good approximation for low-frequency motions [13], [14], and the structural network is often used to study the collective vibrations that are not very sensitive to the sequence specificity due to side chains. [26], [27], [28], [29].

Yet, beyond the backbone-framed protein structure, how to delineate the networks of molecular interactions, what are their differences in comparison to the structural network, and what are the manifestations of the side-chain sequence and dynamical motions? In particular, if the interaction network of side chains could be studied, its properties are expected to differ from that of the backbone. To address these key issues of the sequence-structure-function relationship, a graph-theoretic methodology is devised here to compute the mechanical interactions mediated by side chains and backbone from all-atom molecular dynamics (MD) simulations in an explicit solvent.

**Fig. 1:**
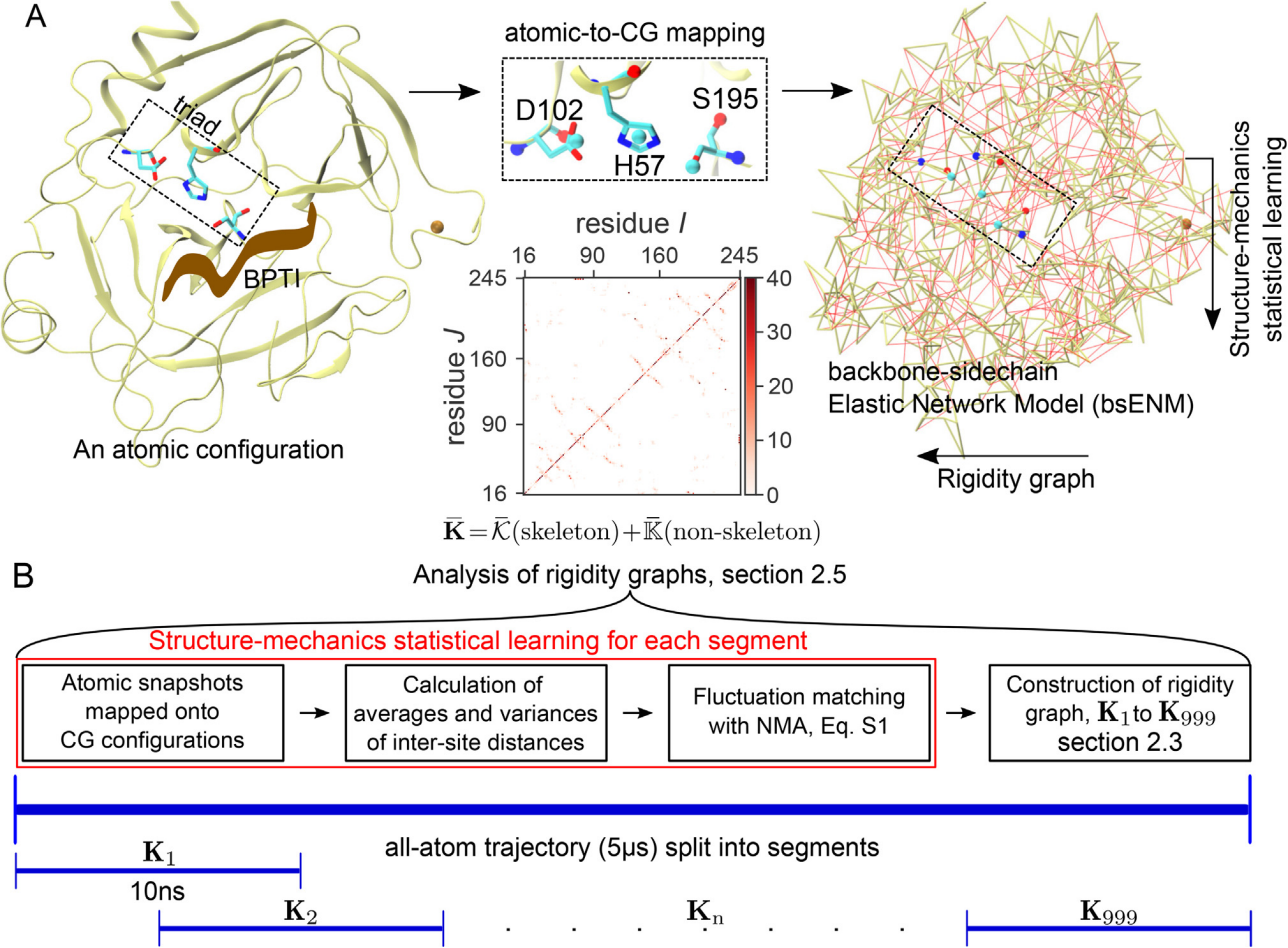
Rigidity graphs of the protein mechanical coupling network statistically learned from all-atom MD, see Materials and Methods for details. (A) A sampled atomic structure is mapped onto the coordinates of backbone and side-chain sites in the coarse-grained (CG) representation of bsENM. Left: a ribbon representation for the atomic structure of RT bound with BPTI. The catalytic triad is highlighted and a zoomed-in view showcases the atomic-to-CG mapping in Table S1. Right: the mapped CG configuration. (B) Dividing the 5 μs all-atom MD trajectory into consecutive 10 ns windows, and the work flow for computing the bsENM parameters of each segment by structure-mechanics statistical learning.

The guiding principle is that given the unique structural position and chemical environment of a protein residue, its couplings with surrounding atoms would assume specific strength during dynamical motions. A mechanical-coupling dynamics perspective is thus taken to unravel the network behaviors of physical interactions. In particular, the elastic parameters in a model of backbone and side-chain nodes connected by harmonic springs, i.e., the backbone-side-chain elastic network model, bsENM, proposed in this work and illustrated in Fig. 1A, is used to represent the effective interaction strengths. Our design of bsENM is to explicitly represent the protein chemical components for resolving the backbone and side-chain contributions in the mechanical coupling network. In particular, the scope of bsENM spring constants is expanded for an unexplored context: to statistically learn the mechanical coupling strengths of backbone and side chains from all-atom MD simulations. The effective elasticities calculated by self-consistent iterations [30], [31], [32] are then used to construct inter-residue graphs. As will be shown later, this bsENM-graph approach can be used to map the protein dynamics into distinct rigidity groupings, namely the backbone-backbone (BB), the backbone-side-chain (BS), and the side-chain-side-chain (SS). Investigating them using spectral analysis allows us to uncover their unique mechanical topologies for comparing with experimental observables such as residue conservation and co-evolution in multiple sequence alignment (MSA), mutation sensitivity, residue flexibility profile, and signals reflecting residue micro-environments. Under this structure-mechanics statistical learning framework, in contrast to the aforementioned topology-only approach, even interaction pairs of similar distance separation can have very different coupling strengths. Using the elastic properties statistically learned from an all-atom MD trajectory as the edge weights in A and D thus offers a new perspective—the rigidity graphs of protein dynamics. To reveal the impact of chemical details, the specifically designed scheme is dividing the bsENM harmonic potentials into (a) skeleton springs as those linking the nearest and second nearest residues and (b) non-skeleton springs as the rest. With A=A(skeletonsprings)+A(non-skeletonsprings) and D=D+D, we first establish that the low-frequency modes of the skeleton Laplacian (L=D-A) are exceedingly insensitive to the variation in strength during protein dynamics. Analysis of mechanical couplings is hence focused on non-skeleton springs. A key finding is that the non-skeleton signless Laplacian (K=D+A) reveals the specific patterns very clearly. The statistically prominent features of K can thus be extracted from the all-atom MD trajectory to reveal the mechanical topologies of different interactions. To illustrate this approach, we choose as case studies a serine protease family member rat trypsin (RT) [33], [34] and PDZ3 (the third PDZ signaling domain) [35], [36], [37] because they both are β-strand rich and have comprehensive data for site-specific mutagenesis and functional activity experiments. In addition, the wealth of sequence information allows the calculation of inter-residue co-evolution from statistical coupling analysis (SCA) [38], [39], [40] or direct coupling analysis (DCA) [41], [42] from MSA and provides complementary insights. The BB (backbone-backbone), SS (side chain-side chain), and BS (backbone-side chain) rigidity graphs can thus be compared with these observables for linking physical interactions with biological functions and evolutionary pressure.

## 2 Materials and methods

The elastic parameters in bsENM ($k_{\mathrm{ij}}$’s) are calculated from an all-atom MD trajectory by matching the fluctuations of inter-site distances. Effectively, this structure-mechanics statistical learning integrates out the other degrees of freedom by self-consistent iteration with normal mode analysis (NMA) [30], [31], [32]. Moreover, mechanical coupling strengths represented by the spring constants are used to construct BB, BS, and SS rigidity graphs. This computational framework is detailed in the following using RT as the example.

### 2.1 All-atom MD simulation

The X-ray structure of BPTI bound RT (PDB ID: 3TGI) is used to construct its all-atom model [33] whereas for PDZ3, the apo X-ray structure (PDB ID: 1BFE) [37] is used. All systems are solvated in orthorhombic dodecahedron TIP3P water boxes and neutralized with NaCl ions at 0.15 M. The CHARMM36 all-atom force field [43] is used to compute the potential energy and the GROMACS software [44] is used for MD runs. The production run for both the RT and PDZ3 systems is at 300 K and 1 atm for 5 μs, during which a snapshot is saved every 1 ps for analysis. The other details are reported in SI.

### 2.2 Structure-mechanics statistical learning of bsENM parameters

With the raw data of protein dynamics generated with full atomic details, the CG sites in bsENM serve to read out the statistics of inter-site elasticity. The goal is to capture the significant mechanical couplings that can survive thermal noise, and the CG sites are thus located where specific molecular interactions are typically observed in the trajectories. In particular, the backbone is represented by two coarse-grained (CG) sites at the amide nitrogen and carbonyl oxygen positions as they are the loci of hydrogen bonding, Fig. 1A. For side chains, a CG site is placed at the position of a representative atom at which specific interactions are formed. For example, the alanine side-chain site is at $C_{\beta }$ and that of lysine is at $N_{\zeta }$. For hydrophobic side-chains, the center of mass of heavy atoms is used. Table S1 lists the details of this atomic-to-CG mapping, which is used to convert each frame in the MD trajectory to a bsENM configuration.

Given a set of bsENM configurations, the spring length $l_{\mathrm{ij}}^{0}$ between sites i and j is their averaged distance. To parametrize $k_{\mathrm{ij}}$, the variance of distance fluctuation, $\langle \delta l_{\mathrm{ij}}^{2}\rangle_{\mathrm{AA}}$, in the all-atom MD data is the targeted value. At each iteration step, NMA of the bsENM gives the predicted distance fluctuation, and $k_{\mathrm{ij}}$ is adjusted to match the targeted value [31], [32]. Since the bsENM springs are connected in the structure, their coupled fluctuations are handled by self-consistent iterations in this fluctuation matching. Other details are reported in SI. To construct the starting model (initial guess) for statistical learning, a cutoff distance $l_{c}$ is used to include a harmonic potential in the bsENM for every inter-site pair with $l_{\mathrm{ij}}^{0}<l_{c}$. The cutoff is thus an adjustable parameter and is determined by scanning the value and comparing the resulting residue RMSF (root-of-mean-squared-fluctuation) and the low-frequency vibrational modes of the statistically learned bsENM with those calculated from all-atom MD. As discussed in Fig. S1, consistent behaviors are observed over a wide range of cutoff values since the bsENM springs are trained to match the all-atom MD target data. The default $l_{c}$ is set to 7.8 Å  as shown in Fig. S1. After convergence of the first round, connectivity trimming followed by another round of fluctuation matching is conducted to prevent having excessive springs in the network; other details are reported in SI. An approximation of bsENM is using a universal value for all springs within the cutoff. This $0^{\mathrm{th}}$-order construction solely relying on the structural network is denoted bsENM_0_. To highlight the effects of chemical details within the native topology, the bsENM statistically learned from all-atom MD is compared with the bsENM_0_ of the same equilibrium structure and $l_{c}$.

### 2.3 Construction of inter-residue rigidity graphs from bsENM

In our graphical representation of inter-residue interaction networks, the edge weights between residue nodes I and J in the adjacency matrix are $k_{\mathrm{IJ}}=\sum_{i\in I,\;j\in J}k_{\mathrm{ij}}$, the sum over the bsENM spring constants linking their CG sites. For bsENM_0_, $k_{\mathrm{ij}}$ is set to 1 for the springs within $l_{c}$. In this graphical theory, the degree matrix components record the total coupling strength of each residue $k_{\mathrm{II}}=\sum_{I\neq J}k_{\mathrm{IJ}}$, or, in the case of bsENM_0_, the residue contact number of CG sites within $l_{c}$. Since bsENM springs can be categorized by the types (backbone or side chain) of their CG sites, the graphs of different rigidity groups can be constructed accordingly. For example, $K^{\mathrm{BB}}$, $K^{\mathrm{BS}}$, and $K^{\mathrm{SS}}$ are the signless Laplacian of the non-skeleton springs in backbone-backbone, backbone-side-chain, and side-chain-side-chain groups, respectively. For $K^{\mathrm{SS}}$, disulfide bond springs are exceedingly strong and are skipped to focus on non-covalent mechanical couplings.

### 2.4 Comparison of rigidity graphs

A protein of N residues can thus have different N×N rigidity graphs that are symmetric and positive-semidefinite or positive-definite. The eigenvectors, which also form an orthonormal basis set, are ordered according to their eigenvalues in a descending order with mode index α. The eigenvectors of a rigidity graph are the specific patterns of mechanical couplings between protein residues. To quantify whether different rigidity graphs have similar behaviors, the following mode-based procedure is developed.

The similarity of graph L with respect to a reference graph R along its mode α is defined as $r_{\alpha }=\max_{\beta }\left|\nu_{\beta }^{L}\cdot \nu_{\alpha }^{R}\right|$, i.e., by finding the eigenvector $\beta_{\max}$ in L that the dot product with mode α in R has the largest magnitude. Here, $\nu_{\beta }^{L}$ and $\nu_{\alpha }^{R}$ are the eigenvectors of the L and R rigidity graphs, respectively. For example, if $r_{\alpha }=1$ for L, the bsENM Laplacian, with respect to $L_{0}$, the bsENM_0_ Laplacian, then L has an identical counterpart as mode α in $L_{0}$. The mode of the compared graph that delivers the phase, $\beta_{\max}$, may not be the same as α in the reference graph, since the respective mode rankings may differ.

### 2.5 Statistical analysis of protein rigidity graphs

With the 5 μs production runs of RT and PDZ3, a single bsENM using the entire trajectory only provides an equilibrium harmonic approximation for structural fluctuations, omitting the potentially interesting and informative fluctuations on the interaction-network level. This limitation is overcome by dividing the trajectory into consecutive 10-ns windows to compute a series of rigidity graphs (see Fig. 1B for an illustration of windowed trajectory segmentation for statistical analysis). The 10-ns window appears to be a reasonably robust window size in terms of extracting mechanical coupling parameters. This is illustrated in Fig. S2, which shows that the distribution of the inter-site interaction strength, $k_{\mathrm{ij}}$’s, appears unchanged in 5-ns, 10-ns, or 20-ns window sizes. Therefore, the coupling network (e.g., the rigidity graph) extracted from a 10-ns window trajectory is used as an element in further statistical analyses.

#### 2.5.1 Mean-modes of fluctuating rigidity graphs

The rigidity graph of a protein fluctuates and evolves over time as the protein experiences thermal fluctuations or interacts with the surrounding molecules. To capture and quantify these fluctuations on the network level using the rigidity graph, we begin by defining “mean-modes” which are to be understood as the average eigenmodes of an otherwise fluctuating rigidity graph. They are computed as follows: From the bsENM of each trajectory window indexed by n, the non-skeleton springs are used to calculate the off-diagonal $k_{\mathrm{IJ}}$ and the diagonal $k_{\mathrm{II}}$ terms in the inter-residue rigidity graph $K_{n}$. Averaging the $K_{n}$ graphs over the temporal segments gives the mean rigidity graph $\overset{¯}{K}$. From the mean rigidity graph $\overset{¯}{K}$, one finds eigenvectors, $\nu_{\alpha^{\prime }}$’s, each of which is a mean-mode with a corresponding eigenvalue $\lambda_{\alpha^{\prime }}$—the coupling strength for the $\alpha^{\prime }$ mean-mode. Note that hereafter a superscripted index such as $\alpha^{\prime }$ is used for variables and functions derived from the mean rigidity graph, $\overset{¯}{K}$.

#### 2.5.2 Content of a mean-mode in an analysis time window

Each mean-mode is a unit vector for the N residues of the rigidity graph, and can be understood as an N-vector mechanical coupling pattern. In this light, in addition to the coupling strength, another important property is how much the pattern of a particular mean-mode is retained in the mechanical coupling network of each of the analysis window. This property is determined by first calculating the mean-mode content in each trajectory window following the description in Section 2.4 as $r_{n\alpha^{\prime }}=\max_{\beta }\left|\nu_{n\beta }\cdot \nu_{\alpha^{\prime }}\right|$, where $\nu_{n\beta }$ is an eigenvector of $K_{n}$ indexed by β. A high mean-mode content of $r_{n\alpha^{\prime }}∼1$ indicates that the pattern of $\nu_{\alpha^{\prime }}$ stays the same in the trajectory window. Averaging the mean-mode content over all trajectory windows then gives the averaged mean-mode content $r_{\alpha^{\prime }}$. It measures the extent to which the eigenvector-pattern is retained throughout the entire all-atom MD trajectory.

#### 2.5.3 Prominent modes of a rigidity graph

With the coupling strength $\lambda_{\alpha^{\prime }}$ and the average content $r_{\alpha^{\prime }}$ defined for a given mechanical-coupling pattern (designated by the mean-mode $\alpha^{\prime }$), we next ask which mean-mode features are most prominent as the protein undergoes dynamical structural fluctuations. For the present work, we define a “prominent mode” as a mean-mode that exhibits both (a) strong strength in the mechanical coupling and (b) high averaged content during the protein dynamics. More quantitatively, for (a), mean-modes exhibiting strong couplings are defined as those showing higher than the upper fence of an empirically defined quantity, $Q_{3}+1.5\times (\mathrm{inter}\text{-}\mathrm{quartile}\;\mathrm{region})$, within the $\left\{\lambda_{\alpha^{\prime }}\right\}$ distribution (see Fig. S3). For (b), mean-modes having high contents during the protein dynamics are based on the cumulative density function (CDF) of $r_{\alpha^{\prime }}$. The cutoff for high-content designation is assigned empirically depending on the type of mechanical coupling. For ${\overset{¯}{K}}^{\mathrm{BB}}$ and ${\overset{¯}{K}}^{\mathrm{BS}}$, only those at top 25% of $r_{\alpha^{\prime }}$ values are considered the candidates of prominent modes, while the empirical percentile cutoff is top 32% for the ${\overset{¯}{K}}^{\mathrm{SS}}$ modes (see Fig. S3 for summary plots). As shown in Figs. S4–S6, the prominent modes of $\overset{¯}{K}$ rigidity graphs exhibit pointed patterns in residue weights ($\nu_{I\alpha^{\prime }}^{2}$), and mechanical hotspots are filtered out as the residues having significant population.

### 2.6 Residue rigidity scores for backbone and side chain

The set of prominent modes in a $\overset{¯}{K}$ rigidity-graph, Π, contains the strong and high-content mechanical coupling patterns during protein dynamics and are potentially important for functional activities. Therefore, key residues in the prominent modes likely have significant biological importance, thereby providing yet further refined chemical specificity. With this insight, the participation of each protein residue in the prominent modes is used to compute a quantitative metric in the mechanical coupling network—the residue rigidity score. In a rigidity graph, we start by finding the characteristic mode $I^{\prime }$ for each residue indexed by I. The protein residue is first associated with a rigidity-graph mode $\pi^{\prime }\in \Pi$ that its weight is the highest, and the $I^{\prime }$ of I is set to $\pi^{\prime }$ if the weight is significant ($\nu_{I\pi^{\prime }}^{2}⩾\nu_{c}^{2}$).

(1) $$I^{\prime }=\left\{\begin{array}{c} \pi^{\prime }=\underset{\beta^{\prime }\in \Pi }{\arg\;\max}\nu_{I\beta^{\prime }}^{2}\;\;\mathrm{if}\;\;\nu_{I\pi^{\prime }}^{2}⩾\nu_{c}^{2}\\ \alpha^{\prime }=\underset{\beta^{\prime }\notin \Pi }{\arg\;\max}\nu_{I\beta^{\prime }}^{2}\;\;\mathrm{if}\;\;\nu_{I\pi^{\prime }}^{2}<\nu_{c}^{2}\text{.} \end{array}\right)$$

In this equation, if residue I does not play a significant role in any of the prominent modes ($\nu_{I\pi^{\prime }}^{2}<\nu_{c}^{2}$), $I^{\prime }$ is one of the rest eigenvectors ($\beta^{\prime }\notin \Pi$) that it has the highest weight. As shown in Figs. S4–S6, the empirical $\nu_{c}^{2}=0.1$ is used to identify the significantly populated residues in a prominent mode. Next, the residue rigidity score in the graph is defined as $\kappa_{I}=\langle r_{I^{\prime }}\rangle \lambda_{I^{\prime }}$, i.e., the mechanical strength weighted by the averaged mean-mode content. If the $I^{\prime }$ of residue I is in Π, its residue rigidity score is high, while if $I^{\prime }$ has a weak strength and/or low averaged content during protein dynamics, $\kappa_{I}$ is low. The residue rigidity score in backbone is defined as $\kappa_{I}^{B}=\max\left(\kappa_{I}^{\mathrm{BB}},\kappa_{I}^{\mathrm{BS}}\right)$, the maximum score the residue delivers though its backbone, and $\kappa_{I}^{\mathrm{BS}}$ is compared only if residue I contributes backbone in the mode. Similarly, the residue rigidity score in side-chain is calculated as $\kappa_{I}^{S}=\max\left(\kappa_{I}^{\mathrm{SS}},\kappa_{I}^{\mathrm{BS}}\right)$, and $\kappa_{I}^{\mathrm{BS}}$ is only considered if residue I participates by its side-chain.

## 3 Results and discussion

With the all-atom MD simulation, structure-mechanics statistical learning, and rigidity graph analysis for both the RT and the PDZ3 proteins, the main text primarily uses RT for introducing the rich and quantitative information made available by our new approach. The two systems illustrate the common general features of sparse mechanical coupling network, scale-free network coupling strengths, hotspots in both backbone and side-chain interaction networks, and the residue rigidity scores during protein-dynamics as a new metric for biochemical functions. In what follows, the mechanical coupling network is analyzed in detail.

### 3.1 The protein mechanical coupling network is sparse

**Fig. 2:**
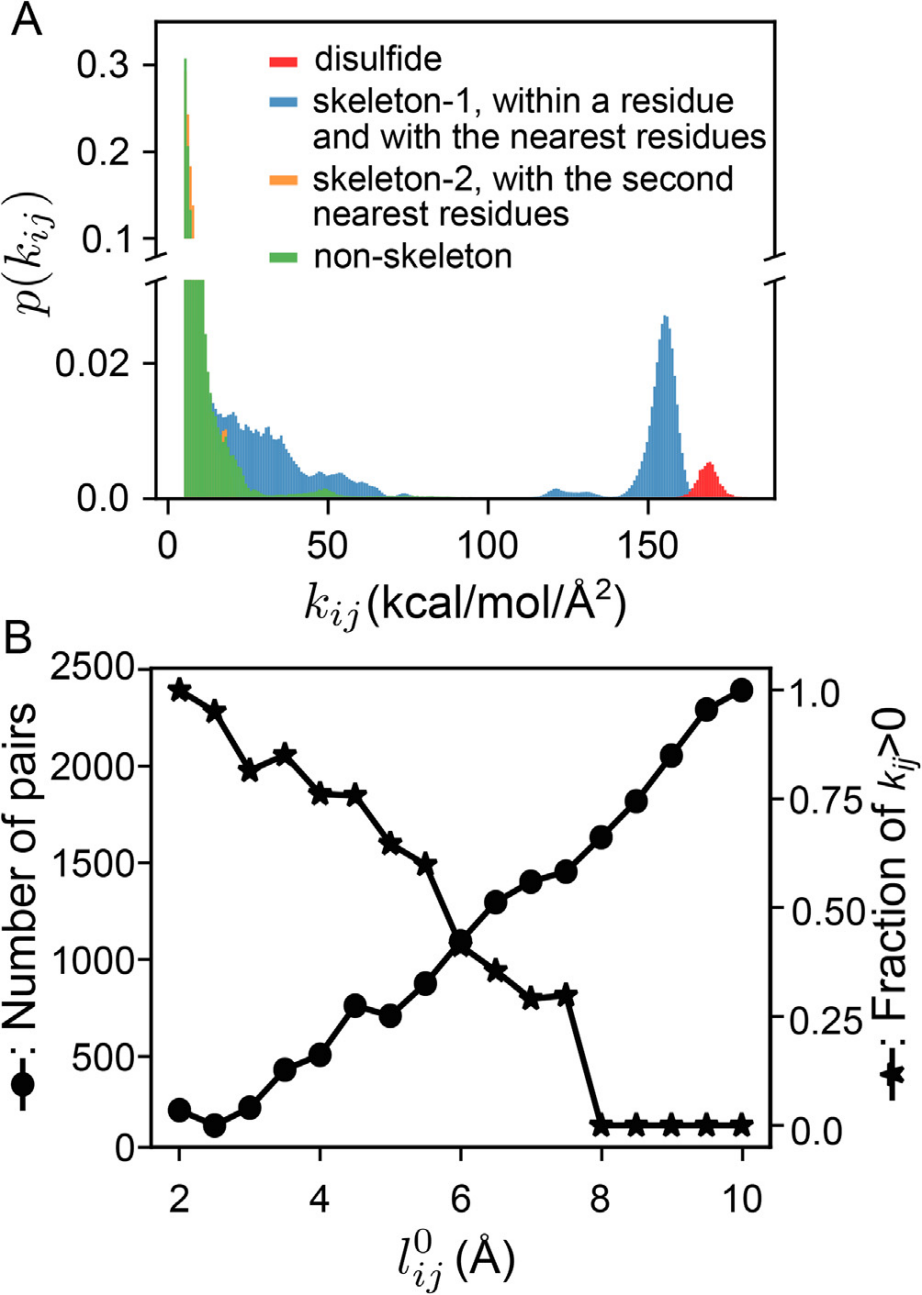
The elastic spring parameters of a bsENM statistically learned from a trajectory window of RT. (A) Normalized histogram of coupling strengths. Between CG sites i and j, $k_{\mathrm{ij}}$ is the coupling strength. Disulfide springs that connect the Sγ atoms of a cysteine pair are very strong. Skeleton-1 includes the springs within a residue and between the nearest residues. The very high strengths in skeleton-1 are peptide bonds, whereas the other springs in this category are on the left. Skeleton-2 is the springs between the second nearest residues. Non-skeleton springs are the rest with disulfide bonds also excluded. (B) In each 0.5 Å  bin of the spring length $l_{\mathrm{ij}}^{0}$, the number of pairs in the protein structure (left) versus the number of springs converging to a non-zero $k_{\mathrm{ij}}$ in the fluctuation matching of a trajectory window (right).

Our structure-mechanics statistical learning with bsENM provides a way to map the chemical details during protein dynamics onto $k_{\mathrm{ij}}$ values. The bsENM calculated from a trajectory segment thus contains a list of springs each with a length $l_{\mathrm{ij}}^{0}$ and a positive-definite elastic constant $k_{\mathrm{ij}}$. The diverse mechanical coupling strengths can be seen in the $k_{\mathrm{ij}}$ distribution from a RT trajectory window, Fig. 2A. The skeleton springs between residues neighbors and disulfide bonds are exceedingly strong and will be discarded later in the analysis of rigidity graphs. In Fig. 2B, the number of inter-site pairs in the protein structure separated by the $l_{\mathrm{ij}}^{0}$ value in each bin is shown with its fraction of springs that converge to $k_{\mathrm{ij}}$>0 after the self-consistent iterations. With increasing $l_{\mathrm{ij}}^{0}$, it can be seen that the fraction of positive-definite springs drops further, and the protein mechanical coupling network is thus progressively sparser than the structural contact network. Sparsity of the mechanical coupling network signifies that the protein fold can afford sequence variations to accommodate different functions, and the network properties of mechanical couplings during protein dynamics can potentially serve to capture the functionally important interactions.

Although the inter-residue Laplacian matrix (L=D-A) of topological contacts is useful in mimicking collective modes, capturing the specific mechanical coupling patterns may call for a different representation. This aspect is illustrated by using the $L_{0}$ of bsENM_0_ from topological contacts (no chemical details) as the reference. For the lowest-frequency modes of L from all-atom MD, the similarity $r_{\alpha }$ being close to 1 with respect to those of $L_{0}$ (Fig. S7A) shows that the collective vibrations in L are indeed insensitive to the chemical details. If considering only the skeleton springs, the L modes are essentially identical to those of $L_{0}$ (Fig. S7A), whereas the modes of L from non-skeleton springs exhibit lower $r_{\alpha }$ values with respect to those of $L_{0}$. Robustness of the low-frequency modes in L thus mostly comes from the skeleton springs.

To better reveal the molecular specificities in inter-residue elasticities ($k_{\mathrm{IJ}}$’s), we seek to the K=D+A rigidity graph. The similarity $r_{\alpha }$ of the K modes with respect to those of $K_{0}$ shows that the lowest-frequency modes of K still exhibit specific behaviors as the $r_{\alpha }$ values are low, Fig. S7. Even for skeleton springs, K exhibit significant differences comparing to $K_{0}$. Furthermore, inspection of the K eigenmodes for non-skeleton springs (Fig. S8) shows that they reveal clear signals for the patterns of non-covalent $k_{\mathrm{IJ}}$’s. Therefore, we employ the K rigidity graph and its BB, BS, and SS parts in the following to uncover the interplay of backbone and side chains in the protein mechanical coupling network.

### 3.2 Protein mechanical coupling networks have scale-free behaviors

**Fig. 3:**
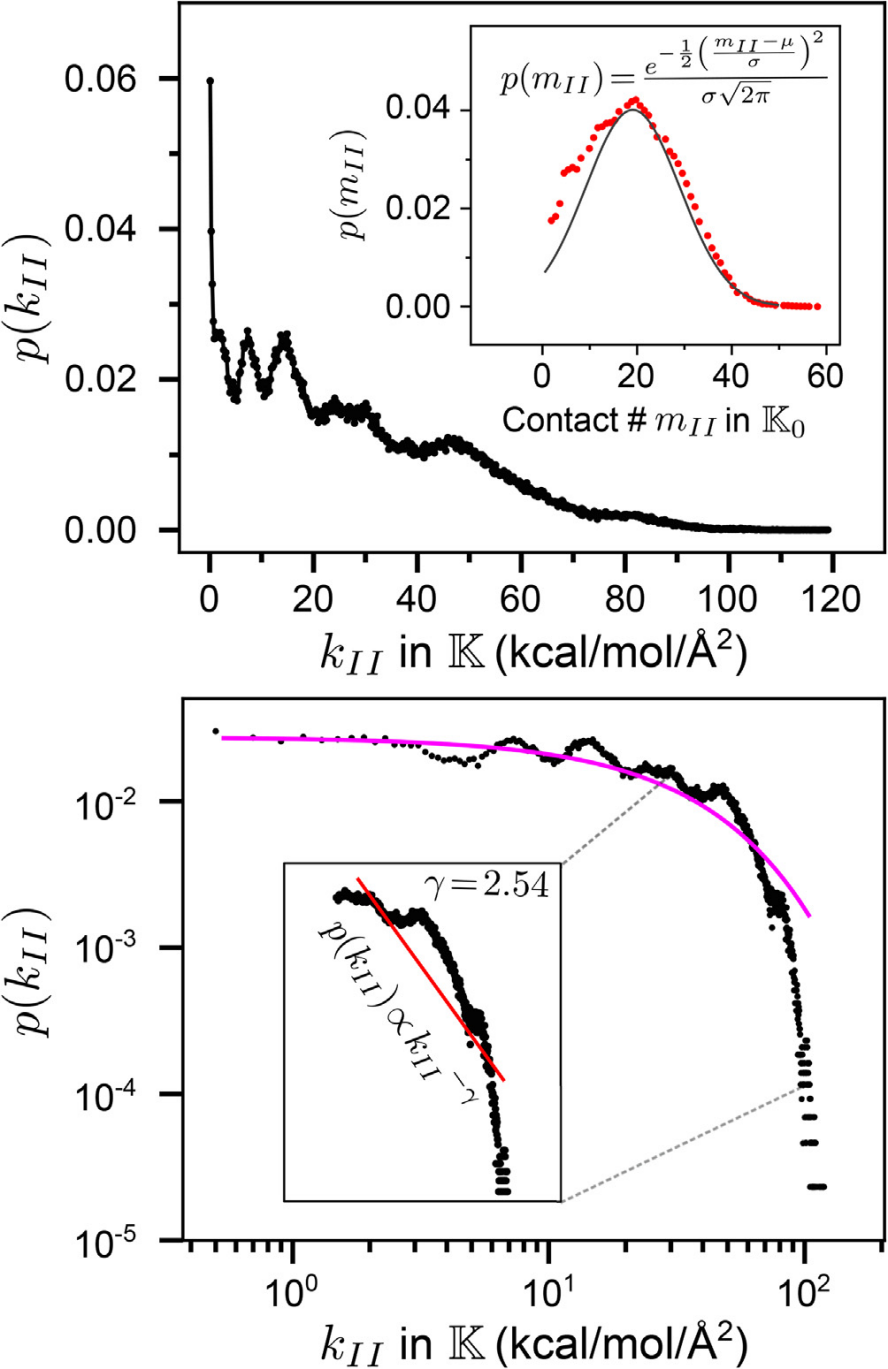
Mechanical couplings during protein dynamics exhibit a heavy tailed distribution and scale-free network behavior. The diagonal components of K (bsENM) and $K_{0}$ (bsENM_0_) in every trajectory window of the 5 μs production run of RT are included. Top panel: $p\left(k_{\mathrm{II}}\right)$, the probability density distribution of $k_{\mathrm{II}}$, the residue coupling strength due to non-skeleton springs in bsENM. Insert: $p\left(m_{\mathrm{II}}\right)$, where the $K_{0}$ diagonal $m_{\mathrm{II}}$ is the residue contact number. It follows a Gaussian distribution given the packing density in the protein structure. Bottom panel: $p\left(k_{\mathrm{II}}\right)$ in the log-log scale. The orange line is the best-fit Lomax distribution. The red line in the insert is a power-law fit with the scaling exponent γ.

The non-skeleton spring constants are used to construct the off-diagonal inter-residue coupling strength $k_{\mathrm{IJ}}$ in K, and the diagonal $k_{\mathrm{II}}$ is the total coupling strength of residue I. In the graphical theory terms of K, $k_{\mathrm{IJ}}$ is the edge weight between the I and J nodes, and $k_{\mathrm{II}}$ is the degree of node I. For a network, the degrees exhibiting power-law scaling in its high-value tale is an indicator of the scale-free property [45], [46], [47]. Such heavy-tailed profiles are due to most nodes having low values, but a small fraction of hotspots exhibits high couplings [48], [49], [50]. As a counter example, residue contact number $m_{\mathrm{II}}$ in the $K_{0}$ graph of the bsENM_0_ is not scale-free given the packing density in a native fold, Fig. 3A. The mechanical coupling network of the bsENM springs statistically learned from all-atom MD, on the other hand, behaves fundamentally different. The probability density $p\left(k_{\mathrm{II}}\right)$ exhibits a long tail and fits quite well with the classical Lomax distribution [51] for heavy-tailed profiles, Fig. 3B. As such, the mechanical coupling strengths during protein dynamics exhibit power-law scaling despite the data having more complicated patterns, and the exponent γ is in the typically encountered range (2<γ<3) of real-world scale-free networks [45], [46], [47], [48], [49], [50]. The protein structural network, however, lacks such scale-free property. The data in Fig. 3 include the bsENM and bsENM_0_ of every 10-ns trajectory window in the 5-μs production run of RT.

The scale-free behavior indicates that structural contacts within similar distances have highly diverse coupling strengths. Our finding of this network property of protein dynamics is consistent with the observation of mutational tolerance [52], [53] that only a certain percentage of mutations would impact the phenotype. Protein rigidity graphs, similarly, have just a fraction of inter-residue edges carrying significant weights, and can potentially serve as the molecular-scale mechanistic basis for mutation sensitivity. Next, the functional connection is analyzed by first focusing on the specific properties of backbone and side-chain mechanical coupling networks.

**Fig. 4:**
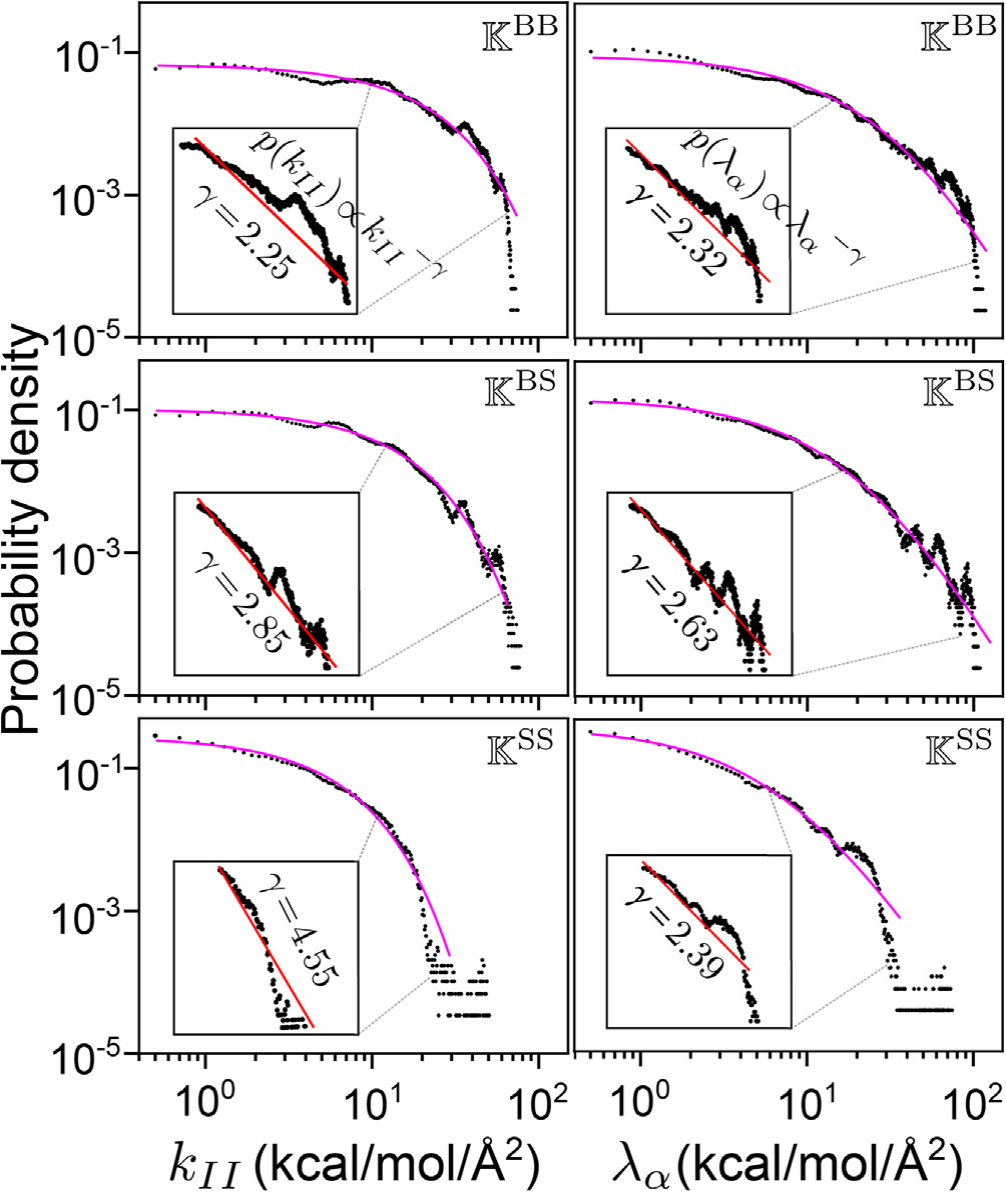
The $p\left(k_{\mathrm{II}}\right)$ (left panels) and $p\left(\lambda_{\alpha }\right)$ (right panels) of $K^{\mathrm{BB}}$, $K^{\mathrm{BS}}$, and $K^{\mathrm{SS}}$ rigidity graphs on a log-log scale. The rigidity graphs of all trajectory windows in the 5 μs production run of RT are used. The orange line is the best-fit Lomax distribution for the heavy-tailed profiles. The red line in the insert is a power-law fit with the scaling exponent γ.

A key advantage in our design of bsENM is that the CG sites are either the backbone or side-chain type. The backbone-backbone, backbone-side-chain, and side-chain-side-chain rigidity graphs can thus be constructed to uncover their separate behaviors in the mechanical coupling network, and $K=K^{\mathrm{BB}}+K^{\mathrm{BS}}+K^{\mathrm{SS}}$. This decomposition of the mechanical coupling network shows that the rigidity graphs of different chemical components all exhibit heavy tails and scale-free behaviors, Fig. 4. Containing the overall weaker non-polar interactions, Fig. S9, the exponent of power-law scaling for the $k_{\mathrm{II}}$ values in $K^{\mathrm{SS}}$ is steeper (γ=4.55), Fig. 4. The high-value outliers in $K^{\mathrm{SS}}$, though, exhibit complicated behaviors that deviate from the simple power-law equation. In an alternative representation by spectral analysis, the rigidity graph eigenvalues ($\lambda_{\alpha }$’s) are the coupling strengths of different modes, and they also exhibit heavy-tailed distributions and power-law scaling. For the $\lambda_{\alpha }$ distribution of $K^{\mathrm{SS}}$, the 2.39 exponent is similar to that of $K^{\mathrm{BB}}$ and $K^{\mathrm{BS}}$, Fig. 4, indicating synergistic combination of inter-residue couplings in the collective modes.

**Fig. 5:**
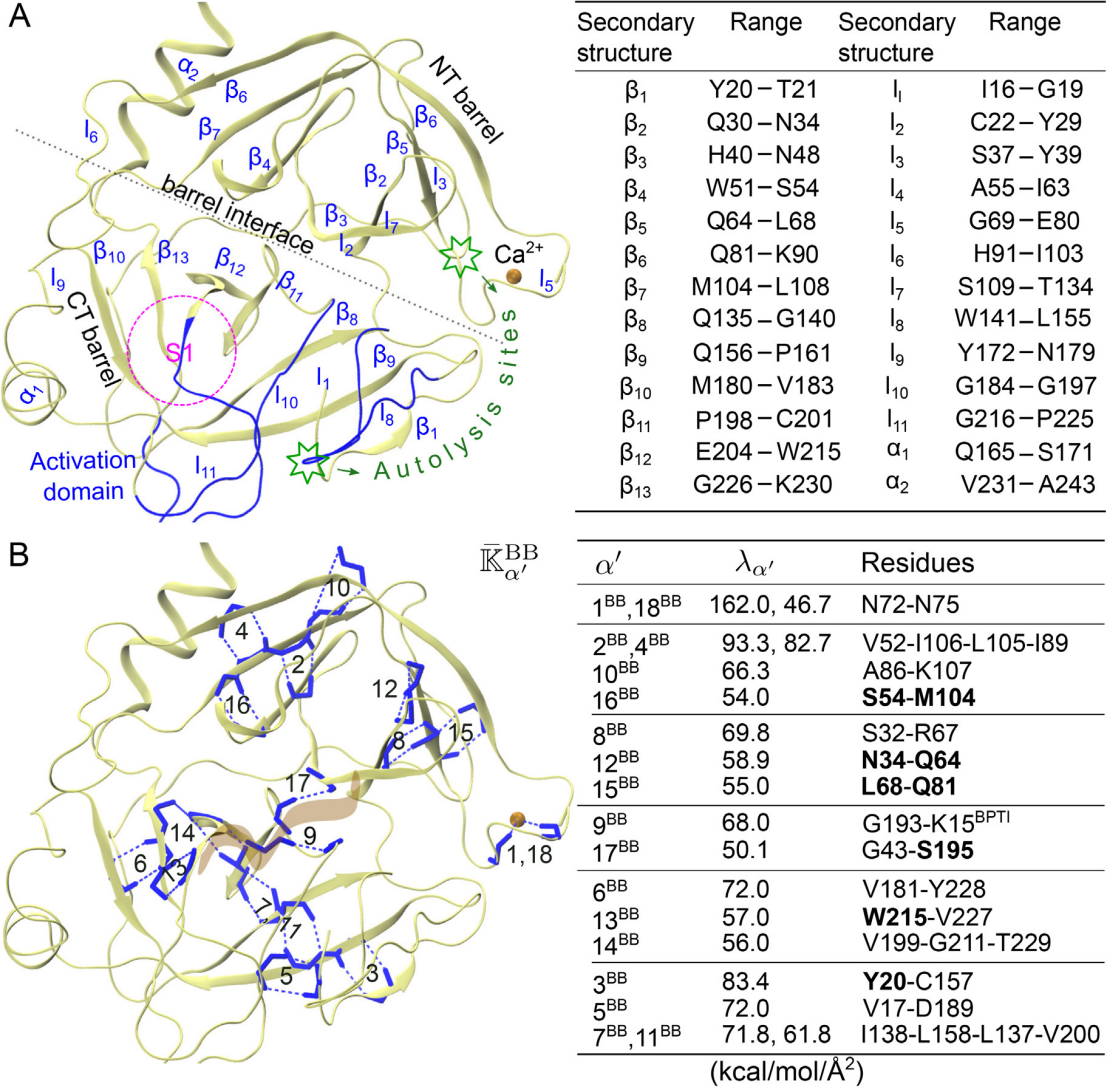
In RT, the prominent backbone-only mechanical couplings during protein dynamics. (A) A ribbon representation the structure and residue composition of secondary structures. (B) Prominent mode residues in ${\overset{¯}{K}}^{\mathrm{BB}}$ are in licorice. The BPTI inhibitor is labeled as brown ribbon. The index, eigenvalue, and residues of prominent modes are listed.

For the protein mechanical coupling networks of a fixed number of nodes (residues) to have scale-free behaviors, their edge weights exhibit high-strength tails as shown in Fig. S9. To analyze the dependence of network properties on chemical differences, the off-diagonal terms of $K^{\mathrm{BB}}$ ($k_{\mathrm{IJ}}$’s) are grouped according to the secondary structure (sheet, helix, and loop) while those of $K^{\mathrm{SS}}$ are divided into polar and nonpolar groups. The $K^{\mathrm{BS}}$ couplings are all polar since backbone is involved. If either residue I or J is not in a helix or sheet, the pair is counted as in loop. The residue composition of RT secondary structures is listed in Fig. 5A. The power-law scalings of these categories are indeed different, and each case has evident higher and/or lower-valued outliers deviating from the simple formula. The α helix $k_{\mathrm{IJ}}$’s are the highest populated in the 5-10 kcal/mol/Å^2^ range, but they do not have any instance of very strong strengths (>15 kcal/mol/Å^2^) and are the least-tailed group, Fig. S9. On the other hand, β sheets have much higher chances of exhibiting exceptional strengths during the dynamical motions and have the lowest γ. Heavy-tailed distributions of strengths are also seen in the BS, BB-loop, and SS-polar couplings. For the nonpolar side chains in RT, its $k_{\mathrm{IJ}}$ tail is shorter and the power-law scaling exponent is higher.

In the 5 μs dynamics of PDZ3, scale-free behaviors of the BB, BS, and SS rigidity graphs are similarly observed, Fig. S10, and the BB-helix and SS-nonpolar couplings are also less heavy-tailed, Fig. S11. The BB-helix $k_{\mathrm{IJ}}$’s, though, have a better fit with the Lomax distribution than those in RT. Interestingly, the polar $K^{\mathrm{SS}}$ components exhibit a much extended tail in PDZ3, and strengths even higher than those of β sheet $K^{\mathrm{BB}}$ show up, Fig. S11, illustrating protein specific behaviors in the mechanical coupling networks.

The results of both RT and PDZ3 show that the mechanical couplings of backbone and side chains have different network properties. To further illustrate this point, specific patterns in the K rigidity graphs of non-skeleton $k_{\mathrm{IJ}}$’s are characterized by spectral decomposition. This analysis also provides the data in identifying the mechanical hotspots based on the contributions of residue backbone and side chains.

### 3.3 Backbone and side chains exhibit specific mechanical hotspots

Graphical analysis of the bsENM quantitatively reveals the backbone and side-chain contributions in the mechanical coupling network. The high-strength tails in the eigenvalue distributions imply that their mechanical coupling patterns are more resistant to thermal noises during protein dynamics. The averaged mean-mode contents during protein dynamics, $r_{\alpha^{\prime }}$, for the eigenmodes of ${\overset{¯}{K}}^{\mathrm{BB}}$, ${\overset{¯}{K}}^{\mathrm{SS}}$, or ${\overset{¯}{K}}^{\mathrm{BS}}$ are calculated following the description in 2.5. The eigenvectors that show a statistically prominent $\lambda_{\alpha^{\prime }}$ and $r_{\alpha^{\prime }}$ over the RT trajectory are then identified in Fig. S3, and their pointed patterns inform the participating residues, Figs. S4–S6. For the 5 μs trajectory of RT bound with BPTI, the rigidity graph includes the inhibitor residues and the Ca^2+^ ion in RT is also treated as an additional residue. It is thus straightforward to adapt the bsENM-graph framework for studying complex protein systems. We focus on the modes of RT residues, and those of BPTI only are not presented.

**Fig. 6:**
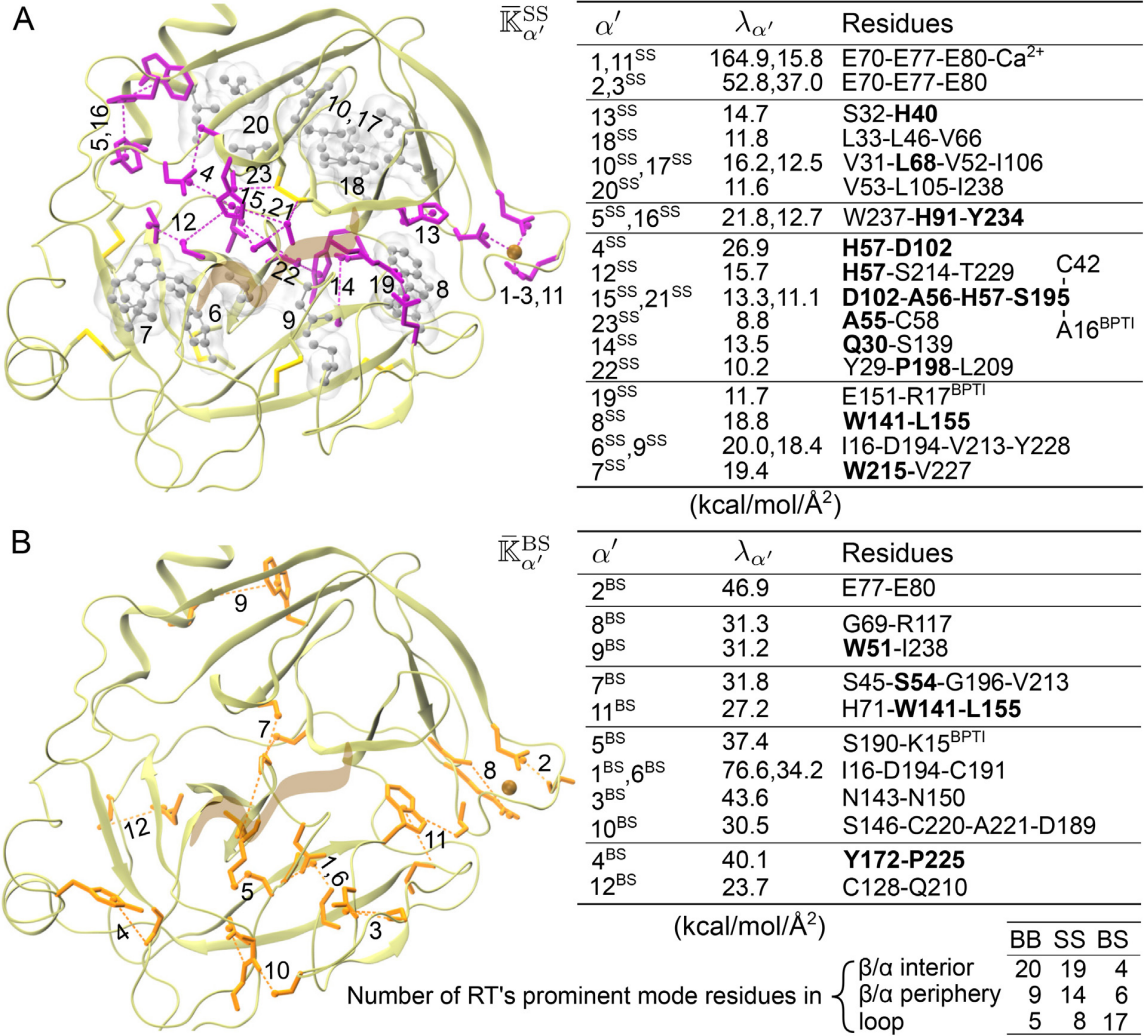
In RT, the prominent mechanical couplings during protein dynamics involving a side chain. The prominent modes in (A) ${\overset{¯}{K}}^{\mathrm{SS}}$ and (B) ${\overset{¯}{K}}^{\mathrm{BS}}$ rigidity graphs. The BPTI inhibitor is in brown ribbon. The RT residues in hydrogen bonds and salt bridges are in licorice and those in hydrophobic couplings are in ball-and-stick. The index, eigenvalue, and residues of prominent modes are listed. The prominent mode residues are hotspots in the mechanical coupling network, and the numbers within a secondary structure, β/α interior, at a secondary structure periphery, β/α periphery, or in a loop are reported.

The trypsin fold of RT containing NT and CT barrels (Fig. 5A) is a useful structural template for therapeutic design [54], [55]. The high-strength ${\overset{¯}{K}}^{\mathrm{BB}}$ eigenvectors indeed have high $r_{\alpha^{\prime }}$ values and Fig. 5B shows the mechanical wiring of the 18 prominent modes. The ${\overset{¯}{K}}^{\mathrm{BB}}$ eigenvector components $\nu_{I\alpha^{\prime }}^{\mathrm{BB}}$ of prominent modes often pick up residue pairs with very strong hydrogen bonds, such as the oxyanion hole G193 coupling to BPTI in ${\overset{¯}{K}}_{9}^{\mathrm{BB}}$, yet more collective patterns ($\alpha^{\prime }$=2, 7, and 14) are also observed. Most of the prominent ${\overset{¯}{K}}^{\mathrm{BB}}$ modes disperse in separate β strand-rich regions, and a noticeable pattern is the strongest coupling locating at the cluster center with few nearby modes containing residues at secondary structure peripheries (edge residues of a β strand or α helix) as boldfaced in Fig. 5B. Out of the 34 hotspot residues in prominent ${\overset{¯}{K}}^{\mathrm{BB}}$ modes, 20 are in secondary structures, 9 are at peripheries, and 5 are in loops, Fig. 6B. The catalytic triad S195, H57, and D102 are within 2-3 residues to β11, β4, and β7, respectively, and are considered at their peripheries. The backbone coupling of S195 with G43 as in ${\overset{¯}{K}}_{17}^{\mathrm{BB}}$ links the NT-barrel and CT-barrel.

Spectral analysis of ${\overset{¯}{K}}^{\mathrm{SS}}$ illustrates a different network topology, Fig. 6A and Fig. S6. In the SS prominent modes, dual-residue patterns often appear at the interface between NT and CT barrels due to very strong hydrogen bonds or salt bridges, such as ${\overset{¯}{K}}_{4}^{\mathrm{SS}}$ between H57 and D102. Polar and nonpolar side chains, though, do not mix in the same prominent modes, demonstrating mechanical coupling separation due to chemical differences. Mostly locating in β strands, the eigenvectors populated by hydrophobic residues tend to involve more mechanically linked partners and can still emerge as prominent modes even the individual $k_{\mathrm{IJ}}^{\mathrm{SS}}$ values may be lower. Comparing to ${\overset{¯}{K}}^{\mathrm{BB}}$, the ${\overset{¯}{K}}^{\mathrm{SS}}$ prominent modes have higher percentages of periphery and loop residues, Fig. 6B. As for ${\overset{¯}{K}}^{\mathrm{BS}}$, the prominent modes are scattered hydrogen bonds primarily in loops, some at secondary structure peripheries, but very few within a secondary structure. In RT, the very strong ${\overset{¯}{K}}^{\mathrm{BS}}$ couplings primarily occur in the CT barrel that contains the activation domain, Fig. 1A and Fig. 6B. Backbone and side-chain mechanical couplings thus exhibit specific patterns in the structure.

The above results of backbone and side chains having distinct mechanical coupling networks provide molecular basis for their separate adjustability as empirically adopted in protein engineering and design [9], [10], [11]. Whether backbone or side chains are more important in shaping the folding funnel is also an unresolved debate [3], [4], [5], [6]. Rather than lumping each residue as a single unit [12], [13], [14], [15], [16], [17], our strategy is explicit representation of backbone and side chains. With their rigidity graphs computed from protein dynamics, this framework provides a refined way for delineating the free-energy landscape around the structure. Next, whether the mechanical couplings would exhibit extended patterns for understanding protein allostery is addressed.

### 3.4 Emergence of extensive mechanical couplings

The RT rigidity graphs reveal that the prominent couplings between RT and BPTI lie in the hydrogen bonding modes ${\overset{¯}{K}}_{9}^{\mathrm{BB}}$, ${\overset{¯}{K}}_{5}^{\mathrm{BS}}$, and ${\overset{¯}{K}}_{19}^{\mathrm{SS}}$ (Fig. 5 and Fig. 6). They are next to several prominent modes within the trypsin fold, including ${\overset{¯}{K}}_{4}^{\mathrm{SS}}$, ${\overset{¯}{K}}_{12}^{\mathrm{SS}}$, ${\overset{¯}{K}}_{17}^{\mathrm{BB}}$ of the triad, and the C42-C58 disulfide bond at the S1’ site. With such spatial arrangement, the prominent modes ${\overset{¯}{K}}_{15}^{\mathrm{SS}}$ and ${\overset{¯}{K}}_{21}^{\mathrm{SS}}$ indeed come out as long-range mechanical couplings containing BPTI A16, S1’ site C42, the catalytic triad, oxyanion hole, A56, and S1 site S214 and T229, Fig. 6A. From the activation domain to active site, Fig. 5B, extensive prominent modes also emerge as ${\overset{¯}{K}}_{1}^{\mathrm{BS}}$, ${\overset{¯}{K}}_{5}^{\mathrm{BS}}$, and ${\overset{¯}{K}}_{10}^{\mathrm{BS}}$, Fig. 6B. A mystery of the serine protease family is that substrate variation or mutation at sites away from the triad still impact the $k_{\mathrm{cat}}/K_{M}$ of cleavage [56], [57], [58], [59], [60]. Our result is a first demonstration that under thermal noise, specific molecular interactions can integrate into significant mechanical signals across distal sites.

During the 5 μs all-atom trajectory of PDZ3, the mechanical hotspots are also captured as the significantly populated residues in the prominent modes, Figs. S12–S15. Similarly, the backbone and side-chain mechanical coupling networks of PDZ3 exhibit different patterns, Figs. S16–S17. Most BB prominent modes are in the β-sandwich with certain extensive patterns like ${\overset{¯}{K}}_{9}^{\mathrm{BB}}$. The prominent BS modes of PDZ3, on the other hand, are more scattered and contain the extensive ${\overset{¯}{K}}_{1}^{\mathrm{BS}}$, ${\overset{¯}{K}}_{3}^{\mathrm{BS}}$, and ${\overset{¯}{K}}_{7}^{\mathrm{BS}}$ that link the β-sandwich and CT-extension. Most of the prominent SS modes in PDZ3 are hydrophobic and rather extensive, while the ${\overset{¯}{K}}_{1}^{\mathrm{SS}}$ salt bridge at the β-sandwich facing CT-extension is exceeding strong, Fig. S17. Similar to RT, the prominent mode residues of PDZ3 ${\overset{¯}{K}}^{\mathrm{SS}}$ have a significantly higher percentage at secondary structure peripheries, such as the two residues of ${\overset{¯}{K}}_{1}^{\mathrm{SS}}$, than those of ${\overset{¯}{K}}^{\mathrm{BB}}$ do. The mechanical hotspots of PDZ3 ${\overset{¯}{K}}^{\mathrm{BS}}$ also very frequently occur at secondary structure peripheries (Fig. S17) rather than in loops as in the case of RT (Fig. 6B). While consistent overall patterns in mechanical coupling networks are observed, the two protein systems exhibit specific features in their prominent modes of rigidity graphs.

At the core of allosteric communication in proteins is the physical interactions that are persistent under thermal noise for connecting distal sites [4], [5], [6]. In attempting to capture such functionally important long-range couplings, many approaches are based on positional covariance [18], [19], [20], structural contacts [21], [22], [23], or sequence co-evolution [38], [39], [40], [41], [42]. However, a fundamental difficulty is that the observed signals do not necessarily correspond to molecular interactions. For example, in using low-frequency vibrational modes to study intra-protein communication, positional fluctuations of unconnected, distal residues can be highly correlated due to the structural topology [12], [13], [14]. From protein dynamics, our computational framework of identifying the prominent modes of K rigidity graphs thus provides a way to capture the molecularly specific patterns that can survive the stochastic fluctuations. In both the RT and PDZ3 protein systems, extensive mechanical couplings composed of physical interactions are identified.

### 3.5 Residue rigidity scores in backbone and side chains during protein dynamics as metrics for biological functions

**Fig. 7:**
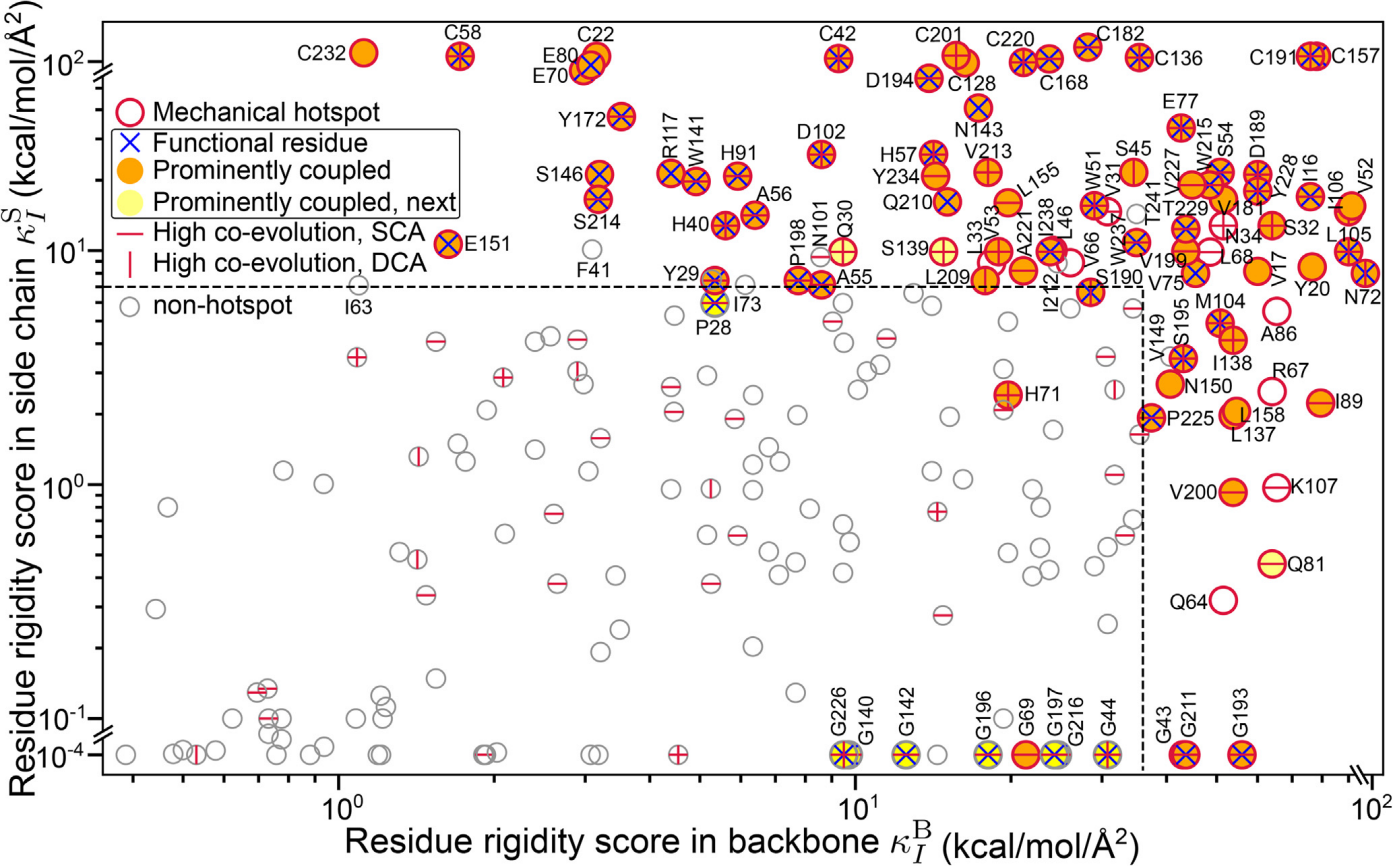
For RT, the residue rigidity score during protein dynamics in backbone ($\kappa_{I}^{B}$, x-axis) and in side chain ($\kappa_{I}^{S}$, y-axis) for residues indexed by I. Since glycine residues do not have a side-chain score, they are provided with a minimal $\kappa_{I}^{S}$ and hence locate at the bottom. Red circles denote mechanical hotspots: the significantly weighted residues in the prominent modes of rigidity graphs. Dashed lines are the hotspot boundary due to ${\overset{¯}{K}}^{\mathrm{BB}}$ and ${\overset{¯}{K}}^{\mathrm{SS}}$ prominent modes, and mechanical hotspots are mostly enclosed in the band except G69, H71, and S190 that contribute backbone to the prominent ${\overset{¯}{K}}^{\mathrm{BS}}$ modes. Labelled  as “functional residue” are those listed in Table S2 that have experimentally verified function or ultra high conservation in MSA. Filled orange as “prominently coupled” are the mechanical hotspots of a single rigidity graph mode that contain least one functional residue. Filled yellow as “prominently coupled, next” are the hotspots next to a functional residue in sequence or non-hotspot functional residues next to a hotspot. A residue is labeled  or  for high co-evolution in SCA or DCA, respectively. Grey circles are non-hotspot residues.

Being the strong mechanical couplings persistent through protein dynamics, the prominent modes of ${\overset{¯}{K}}^{\mathrm{BB}}$, ${\overset{¯}{K}}^{\mathrm{BS}}$, and ${\overset{¯}{K}}^{\mathrm{SS}}$ likely have important implications in biological functions. They also represent the routes through which protein backbone and side chains are wired in the mechanical coupling network. Based on this mechanistic insight, we propose to quantify the biological importance of residues by deducing the residue rigidity scores in ${\overset{¯}{K}}^{\mathrm{BB}}$, ${\overset{¯}{K}}^{\mathrm{BS}}$, and ${\overset{¯}{K}}^{\mathrm{SS}}$. Since their eigenvectors exhibit pointed patterns, each residue is specifically populated in few modes. The residue rigidity score for I in a particular rigidity graph thus comes from the characteristic eigenvector $I^{\prime }$ that the residue is most representative (see Eq. 1 discussed in 2.6). The residue rigidity score $\kappa_{I}$ is then the mechanical strength weighted by the averaged content of mode $I^{\prime }$ during protein dynamics, $\langle \;r_{I^{\prime }}\rangle \lambda_{I^{\prime }}$. By putting together the results of ${\overset{¯}{K}}^{\mathrm{BB}}$, ${\overset{¯}{K}}^{\mathrm{BS}}$, and ${\overset{¯}{K}}^{\mathrm{SS}}$, the residue rigidity score in backbone, $\kappa_{I}^{B}$, is the largest mechanical contribution from the backbone of residue I, and $\kappa_{I}^{S}$ is that from its side chain. Therefore, if residue I plays a significant role in a prominent mode in ${\overset{¯}{K}}^{\mathrm{BB}}$, ${\overset{¯}{K}}^{\mathrm{BS}}$, and/or ${\overset{¯}{K}}^{\mathrm{SS}}$, it would have high $\kappa_{I}^{B}$ and/or $\kappa_{I}^{S}$. On the other hand, if a residue only appears in modes having low strength and/or averaged content, it would have low residue rigidity scores. The residue rigidity scores of RT backbone and side chains in the all-atom MD simulation are shown in Fig. 7; glycine residues listed in the bottom are provided with a minimal $\kappa_{I}^{S}$ as they do have side chain and hence the corresponding score.

In contributing to the mechanical coupling network, Fig. 7 illustrates that the protein amino acids have diverse residue rigidity scores in backbone and side chains. Mechanical hotspots are the significantly populated residues in the prominent mode of rigidity graphs as defined in 2.6, and the dashed lines in Fig. 7 are the boundaries due to ${\overset{¯}{K}}^{\mathrm{BB}}$ and ${\overset{¯}{K}}^{\mathrm{SS}}$. The general importance of the trypsin fold in RT has led to a variety of functional characterization including mutagenesis at different sites. Combining the residues with experimentally verified function and ultra high conservation in MSA provides the functional residues of RT as summarized in Table S2 and marked on Fig. 7. It can be seen that the mechanical hotspots cover most of the functional residues as well as their prominently coupled associates in the rigidity graphs. Although glycine residues do not have a side chain are often flexible, few of them still emerge as mechanical hotspots through backbone such as the oxyanion hole G193. Even though several functional glycines are not in a prominent mode of the rigidity graphs, their signature in the networks is being sequence neighbors of mechanical hotspots, Fig. 7. While the rigidity scores are based on non-skeleton springs, sequence neighbors are prominently coupled through skeleton connections. Therefore, the ”prominently coupled, next” category that the aforementioned glycine residues reside also includes the mechanical hotspots that are next to functional residues. Moreover, many mechanical hotspots exhibit strong signals in SCA and/or DCA [39], [40]; Table S3 summarizes the residues having high co-evolution as marked in Fig. 7. For example, the residues in the aforementioned ${\overset{¯}{K}}_{15}^{\mathrm{SS}}$ and ${\overset{¯}{K}}_{21}^{\mathrm{SS}}$ prominent modes that form a spatially extensive set of mechanical couplings are all in a SCA sector, which also includes the mechanical hotspots in ${\overset{¯}{K}}_{1}^{\mathrm{BS}}$ (Fig. 6 and Table S3).

From the 5 μs all-atom MD data of PDZ3, the mechanical hotspots covering most of its functional residues [38] is also observed in its $\kappa_{I}^{B}$-$\kappa_{I}^{S}$ plot, Fig. S18. The mechanical hotspot F325 important for substrate recognition has been shown to co-evolve with another hotspot H372 at a distal site with A347 and L353 on the communication pathway, but the underlying physical interactions are unclear [61]. As a molecular mechanism for this SCA-based prediction, Fig. S17 shows that F325 is in a cluster of prominent hydrophobic modes (${\overset{¯}{K}}_{2-5,7}^{\mathrm{SS}}$) that together with ${\overset{¯}{K}}_{6}^{\mathrm{BS}}$ mechanically link the residue with H372. One of the mechanical hotspots in these modes is I341, which has been proposed as an alternative route in a thermal-diffusion MD study [62]. Our rigidity graph analysis based on all-atom MD simulations thus offers a unified mechanistic picture for the various data on intra-PDZ3 communication.

Although most of the characterized residues are in the β-sandwich [38] of PDZ3, several mechanical hotspots are found at the interface contacting CT-extension. For example, D357 coupling to Y392 at the interface in ${\overset{¯}{K}}_{1}^{\mathrm{BS}}$ is the most conserved residues in the β-sandwich [61]. It would thus be valuable to specifically examine the functional roles of such residues in inter-domain communication [63], [64]. Overall, the residue rigidity scores in backbone and side chains are very useful metrics for the functional importance of RT and PDZ3 sites. This establishment opens a new door for using molecular simulation to study the mechanistic basis of biological activities and evolutionary restraints.

## 4 Conclusions

Given a protein fold, residue contact numbers center around a value due to the packing density. But then, what is the manifestation of sequence specificities in the structure? This question is addressed here by developing a bsENM with structure-mechanics statistical learning to compute the elastic parameters from 5 μs all-atom MD simulation in explicit solvent. To analyze the network behaviors of the complicated molecular interactions in structural fluctuations, the newly devised graph-theoretic framework introduces the concept of protein rigidity graphs. A key discovery is that the chemical details during protein dynamics render scale-free network properties in the mechanical coupling strengths of both backbone and side chains. In the nano-scale network of a single protein, exhibition of small-world-like features has not been shown to the best of our knowledge. The significantly populated residues in the statistically prominent modes of rigidity graphs are thus recognized as mechanical hotspots.

Furthermore, our bsENM-graph approach enables the direct comparison of backbone and side-chain mechanical couplings to accentuate their differences. Such outcomes point to an important notion that protein residues have diverse combinations of backbone and side-chain contributions to the mechanical coupling network as seen in the $\kappa_{I}^{S}$-$\kappa_{I}^{B}$ plot of RT (Fig. 7) and PDZ3 (Fig. S18). Encouragingly, functional residues of the two protein systems are largely mechanical hotspots themselves or next to one in sequence as for glycine. While most functional residues have high residue rigidity scores for their side chains, some also have prominent backbone couplings as in the top-right corner of Fig. 7 and Fig. S18. Only a specific set of sites having top residue rigidity scores in both the side chains and backbone indicates sophisticatedly tuned interaction network and has implications in shaping the folding funnel and in rendering proper conformational flexibilities for function. Another finding is that a significant portion of side-chain related mechanical hotspots locate at secondary peripheries (Fig. 6 and Fig. S17), i.e., the edges of foldons [65], and are potentially important factors in adopting the foldon inspired models [65], [66] for protein folding. Although the all-atom MD data depend on empirical force fields, specific conditions, and duration, the structure-mechanics statistical learning and graphical analysis schemes as well as the concepts therein can be readily applied to different cases. The mechanical hotspots and prominent rigidity graph modes identified in molecular simulation also bear similarities with the co-evolution in MSA, which also suffers from statistical noises. For RT, the physically contiguous residues in ${\overset{¯}{K}}_{15,21}^{\mathrm{SS}}$ and ${\overset{¯}{K}}_{1,10}^{\mathrm{BS}}$ also exhibit prominent co-evolution signals in MSA as a single sector, Fig. 6 and Table S3. For PDZ3, the multiple prominent modes involving F325 (${\overset{¯}{K}}_{2-5}^{\mathrm{SS}}$) primarily involve sector residues, Fig. S18. This work thus suggests a molecular mechanism for the coupled sequence variation due to evolutionary restraints, that it be associated with having prominent patterns in the protein mechanical coupling network.

## CRediT authorship contribution statement

**Nixon Raj:** Conceptualization, Methodology, Software, Writing - original draft. **Timothy Click:** Methodology, Software. **Haw Yang:** Conceptualization, Writing - review & editing. **Jhih-Wei Chu:** Conceptualization, Methodology, Software, Writing - original draft, Writing - review & editing, Supervision.

## Declaration of Competing Interest

The authors declare that they have no known competing financial interests or personal relationships that could have appeared to influence the work reported in this paper.
