# Supplementary material for: Mechanical couplings of protein backbone and side chains exhibit scale-free network properties and specific hotspots for function
Source: Comput Struct Biotechnol J. 2021 Sep 8;19:5309–20. doi: 10.1016/j.csbj.2021.09.004 (PMC8554173; doi:10.1016/j.csbj.2021.09.004)
Supplement: Supplementary file 1 [file mmc1.pdf]

Supplemental data for:
Mechanical Couplings of Protein Backbone and Side Chains Exhibit Scale-free Network Properties and Specific Hotspots for Function

Nixon Raj[a], Timothy Click[a], Haw Yang[e], Jhih-Wei Chu[a,b,c,d,*]

[a]*Institute of Bioinformatics and Systems Biology, National Yang Ming Chiao Tung University, 75 Bo-Ai Street, Hsinchu 30010, Taiwan, ROC*
[b]*Department of Biological Science and Technology, National Yang Ming Chiao Tung University, 75 Bo-Ai Street, Hsinchu 30010, Taiwan, ROC*
[c]*Institute of Molecular Medicine and Bioengineering, National Yang Ming Chiao Tung University, 75 Bo-Ai Street, Hsinchu 30010, Taiwan, ROC*
[d]*Center for Intelligent Drug Systems and Smart Bio-devices (IDS^{2}B), National Yang Ming Chiao Tung University, 75 Bo-Ai Street, Hsinchu 30010, Taiwan, ROC*
[e]*Department of Chemistry, Princeton University, Princeton NJ 08544, USA*

1. All-atom MD simulation

The protein systems simulated in this work are rat trypsin (RT) [1] and the PDZ3 domain of postsynaptic density protein 95 (PSD-95) [2] using the CHARMM36 protein force field[3]. After the solvation and ionization of RT and PDZ3, these protein systems are energy minimized using steepest descent and heated to 300K by Langevin dynamics using GROMACS[4], which is also used for equilibration and production runs. The short range cutoff radius for van dar Waals interactions and short-range particle-mesh Ewald (PME) terms of electrostatics are 12 Å with a switching function starting at 10 Å. Following heating, equilibration at 1.013 bar and 300 K is conducted using the Berendsen barostat and Langevin thermostat. During the first ns of the NPT equilibration, harmonic restraints of 1000 kJ/mol/nm^2 are applied to the C_α positions resolved in the X-ray reference structure, which are removed in the second ns of NPT equilibration. The production run for 5 μs is then conducted at 1.013 bar and 300 K using the Parrinello-Rahman barostat and Langevin thermostat. A snapshot is saved every 1 ps for analysis. Throughout dynamics simulations, all bonds involving hydrogen atoms are constrained at their equilibrium lengths using LINear Constraint Solver (LINCS)[5].

2. Statistical learning of bsENM parameters from all-atom MD

Each amino acid backbone in bsENM contains two coarse grain (CG) sites at amide nitrogen and carbonyl oxygen positions in the atomic structure, Fig.1A. Glycine only has the two backbone sites. For the side-chain CG site, the position is calculated according to the heavy atom(s) mediating specific interactions. Aromatic residues, for example, the side-chain CG site is set to the center of mass of the ring atoms. The atomic-to-CG mapping of the 3-site bsENM is reported in Table S1.

Each frame of an MD trajectory is mapped to a bsENM configuration accordingly. The averaged distance between CG sites i and j in a trajectory segment is set as their spring length l^0_{ij}, and the variance of distance fluctuations, $\langle \delta l^2_{ij} \rangle_{\mathrm{AA}}$, is also calculated as the target data for parametrizing the elastic coupling strength k_{ij}. Given a cutoff distance l_c, all springs with $l^0_{ij} \leq l_c$ are included in the bsENM, and the determination of l_c is discussed later. By performing normal mode analysis (NMA) of the bsENM at the MD temperature for an iteration step (n), $\langle \delta l^2_{ij} \rangle^{(n)}_{\mathrm{NMA}}$ can be calculated from the eigenvalues and eigenvectors of bsENM (Eq. (62) in [6]). The bsENM inter-site distance fluctuations are then

*Corresponding author
Email address: jwchu@nctu.edu.tw (Jhih-Wei Chu)

matched to the targeted data by adjusting the spring constants:

$$k_{ij}^{(n+1)} = k_{ij}^{(n)} - \alpha \left(\frac{1}{\langle \delta l_{ij}^2 \rangle_{\mathrm{NMA}}^{(n)}} - \frac{1}{\langle \delta l_{ij}^2 \rangle_{\mathrm{AA}}} \right). \tag{S1}$$

Since the springs are inter-connected, these elastic coupling strengths are self-consistently iterated, and α is the learning factor. Only non-negative values are allowed for k_{ij} by setting the value to zero if negativity occurs and convergence of fluctuation matching generally takes several hundred iteration steps. Typical convergence profiles for bsENM springs during the self-consistent iteration are illustrated in Fig. S2B. The total number of springs for the bsENM in the number that k_{ij} takes a positive value.

To determine l_c, its value is scanned and the profile of residue RMSF (root-of-mean-squared-fluctuation) from the fluctuation matched bsENM is compared with that of all-atom MD. Furthermore, the low-frequency vibrational modes of bsENM and the quasi-harmonic modes [6] from the all-atom MD data are also compared for different l_c values. The idea is that given similar performance, a shorter cut-off radius is preferred. As shown in Fig. S1A, the all-atom RMSF profile is prominently reproduced by bsENM with fluctuation matching over a significant range of l_c, and consistent behaviors are also observed for the similarity with low-frequency quasi-harmonic modes from all-atom MD, Fig. S1B. The default l_c is thus set to 7.8 Å. Since the coupling strengths calculated from all-atom MD generally decrease with l_{ij}^0, many springs longer than 6 Å converge to low k_{ij} values. To prevent excessive springs in the network, we trim a spring if the two sites it connects both have lower RMSF comparing to the atomistic MD values. This refinement of bsENM is conducted from the longest spring down to 6.0 Å, followed by another round of fluctuation matching. This length cut-off for trimming is also determined by scanning its value for the range of consistent behaviors.

3. Statistical analysis of protein rigidity graphs

As discussed in the main text, the single harmonic potential provided by bsENM is oversimplified for encompassing the μs time-scale structural variation in all-atom MD. An effective approach to overcome this limitation is dividing the total duration into overlapping segments of a shorter time-scale such as 10 ns shown in Fig.1B and compute an individual bsENM for each block of the trajectory data. For example, if disulfide bonds were included in the side-chain-side-chain rigidity graph, $\overline{\mathbb{K}}^{\mathrm{SS}}$, they would be the modes of highest eigenvalues among $\lambda_{\alpha'}$ and they are present through out the entire all-atom MD trajectory. Therefore, disulfide bonds would be trivially picked as prominent modes. The coupling strength, k_{ij} of disulfide bonds are also consistent with the all-atom force field between S_γ atoms, Table S2. In such S_γ-S_γ modes of the rigidity graph, the two participating cysteine residues exhibit large amplitudes in the eigenvector of $\boldsymbol{v}_{\alpha'}$ while the other residues have close-to-zero components. This behavior indicates that protein rigidity graphs constructed basing on bsENM spring constants can indeed capture the specific inter-residue couplings during protein dynamics. To better reveal the coupling patterns due to non-covalent interactions, we study protein rigidity graphs by focusing on non-skeleton springs between residue pairs $(I, I \pm 3)$ and beyond, and disulfide bonds are excluded from $\overline{\mathbb{K}}^{\mathrm{SS}}$. In such cases, the eigenvectors of $\overline{\mathbb{K}}^{\mathrm{BB}}$, $\overline{\mathbb{K}}^{\mathrm{SS}}$, and $\overline{\mathbb{K}}^{\mathrm{BS}}$ also exhibit the general behavior of specific spiky patterns, and the residues having large components in these modes can be read out to identify the specific molecular interaction, Fig. S4-S6 and Fig. S13-S15.

An example dataset and the code to reproduce the results are linked to “Mendeley Data” repository. The data and code can be downloaded from the link: https://data.mendeley.com/datasets/mv3tzvgjjz

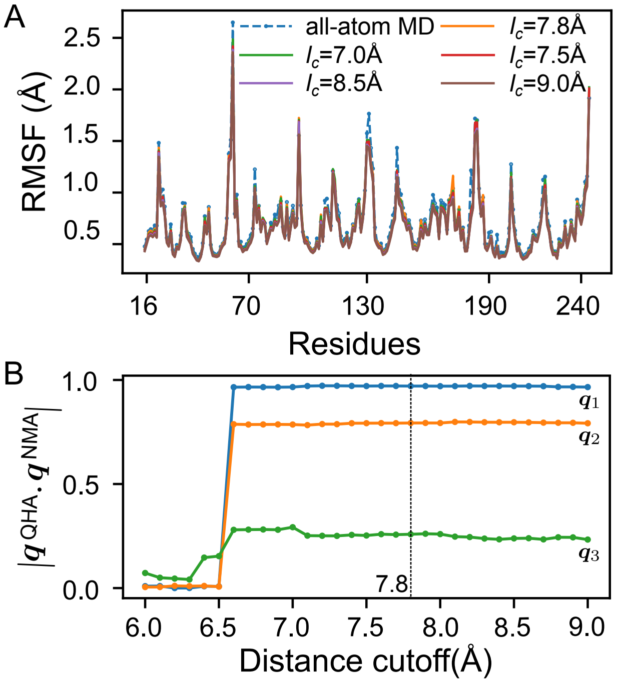

Figure S1: Determination of the l_c cutoff for constructing the initial spring list of bsENM for structure-mechanics statistical learning. (A) Given a l_c value, the residue RMSF (root-of-mean-squared-fluctuation) profile predicted based on the fluctuation-matched bsENM with normal mode analysis and that calculated from the all atom MD trajectory using quasi-harmonic analysis. (B) Comparison of the low-frequency vibrational modes of bsENM and the quasi-harmonic modes of the corresponding all-atom MD trajectory. The dot products of mode eigenvectors are plotted with l_c. The results show a wide range of l_c values giving consistent behaviors.

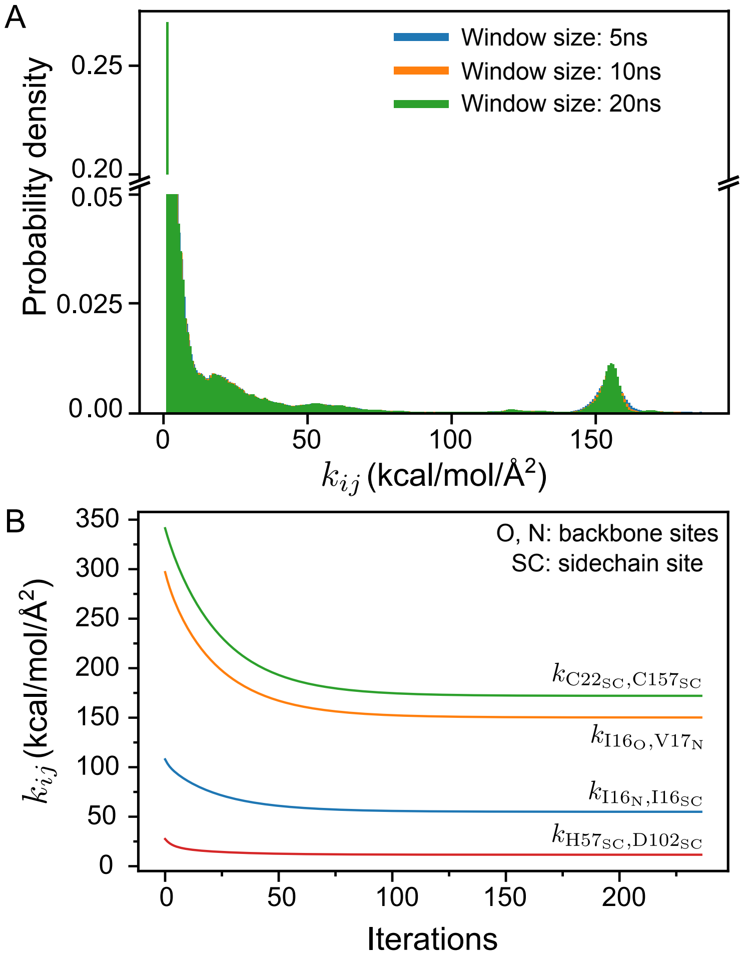

Figure S2: (A) The histograms of k_{ij} in the structure-mechanics statistical learning of bsENM from the all-atom MD trajectory of RT using a 5, 10, or 20 ns window size (B) Example of convergence for the spring constants (k_{ij}'s) in the fluctuation matching. Four representative CG site pairs with different strengths are shown.

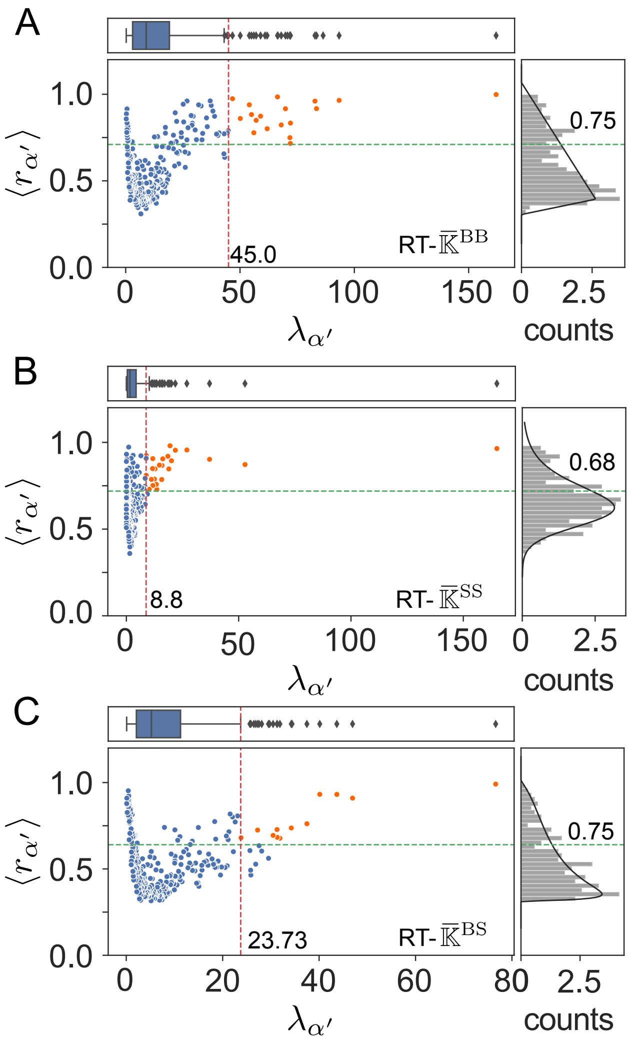

Figure S3: For RT, identification of the statistically prominent modes in a $\overline{\mathbb{K}}$ rigidity graph during protein dynamics from the distribution of eigenvalue ($\lambda_{\alpha'}$) and averaged mean-mode content ($\langle r_{\alpha'} \rangle$). (A) $\overline{\mathbb{K}}^{\mathrm{BB}}$, (B) $\overline{\mathbb{K}}^{\mathrm{SS}}$, (C) $\overline{\mathbb{K}}^{\mathrm{BS}}$. The box plot on the top shows the distribution of $\lambda_{\alpha'}$ and that on the right shows the distribution of $\langle r_{\alpha'} \rangle$. The red dotted lines indicate the cutoff of $\lambda_{\alpha'}$ for an exceptionally strong mode. The green dotted lines indicate the cumulative density cutoff used for identifying modes with high $\langle r_{\alpha'} \rangle$ from the distribution fit. The orange markers indicate the identified prominent modes.

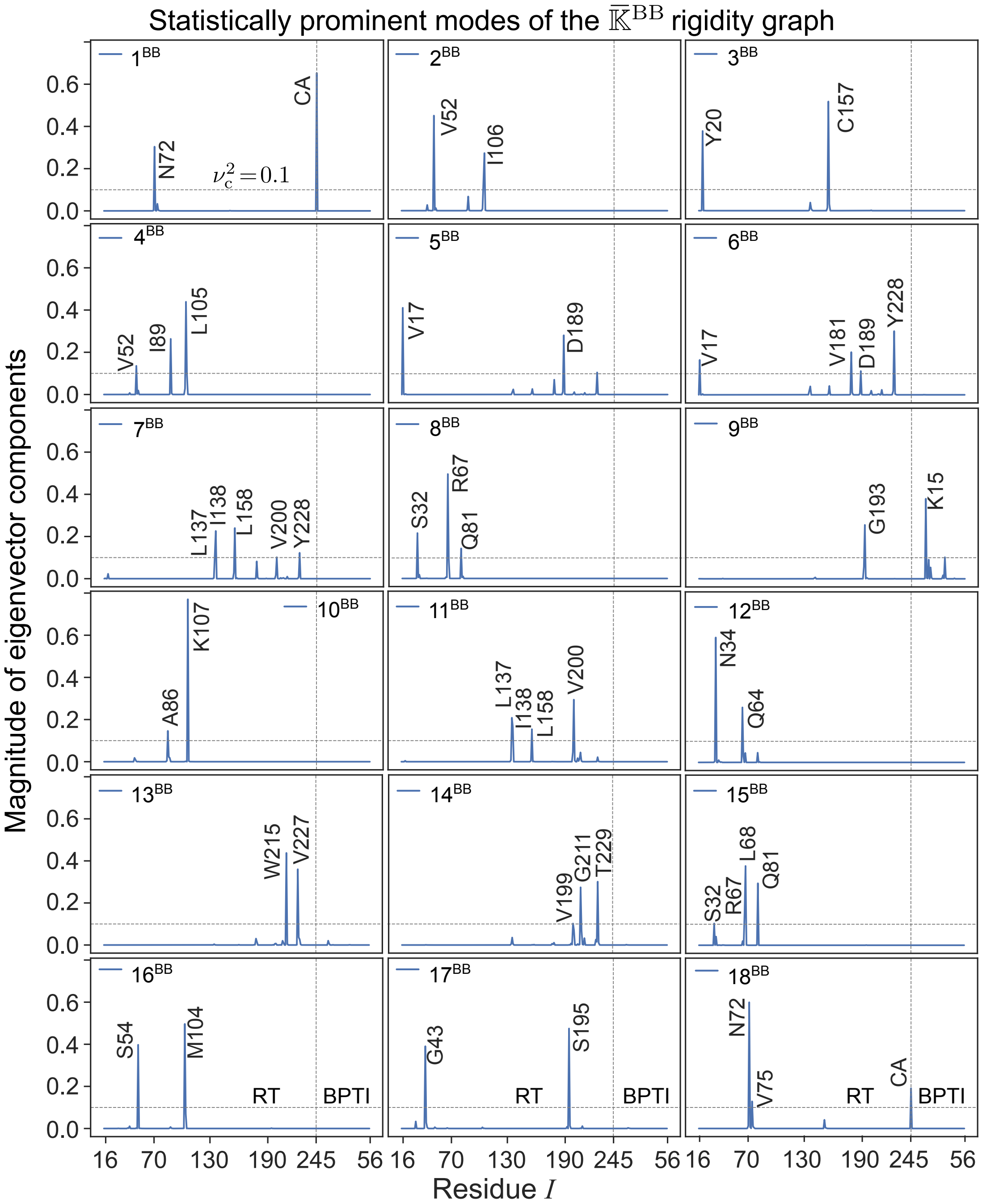

Figure S4: Prominent modes of the RT $\bar{\mathbb{K}}^{\mathrm{BB}}$ rigidity graph. The magnitude of the eigenvector component for each residue is shown. The dotted horizontal line is the magnitude cutoff for identifying the significantly contributing residues of each eigenvector. The dotted vertical line separates the eigenvector components for RT and BPTI residues. The residues are indexed based on the numbering in PDB ID: 3TGI.

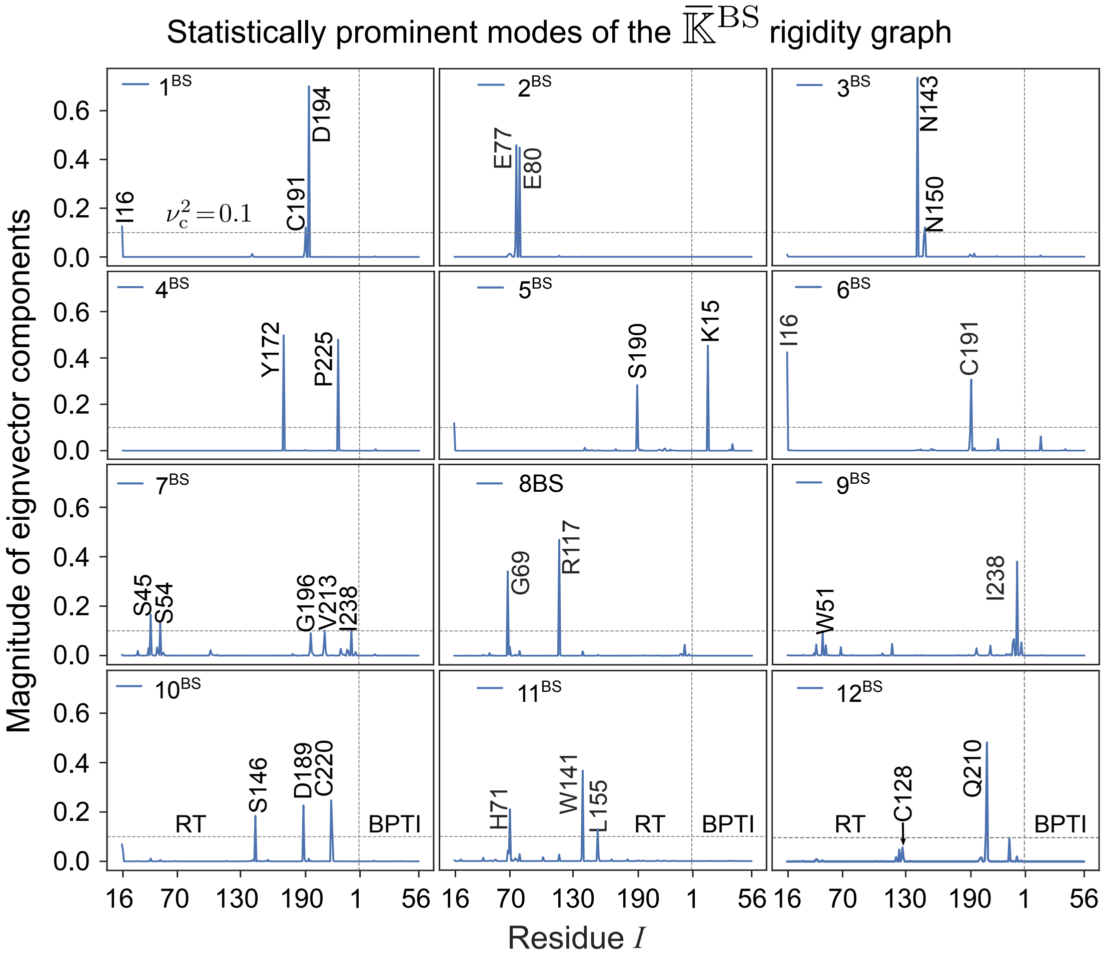

Figure S5: Prominent modes of the RT $\bar{\mathbb{K}}^{\mathrm{BS}}$ rigidity graph. The magnitude of the eigenvector component for each residue is shown. The dotted horizontal line is the magnitude cutoff for identifying the significantly contributing residues of each eigenvector. The dotted vertical line separates the eigenvector components for RT and BPTI residues. The residues are indexed based on the numbering in PDB ID: 3TGI.

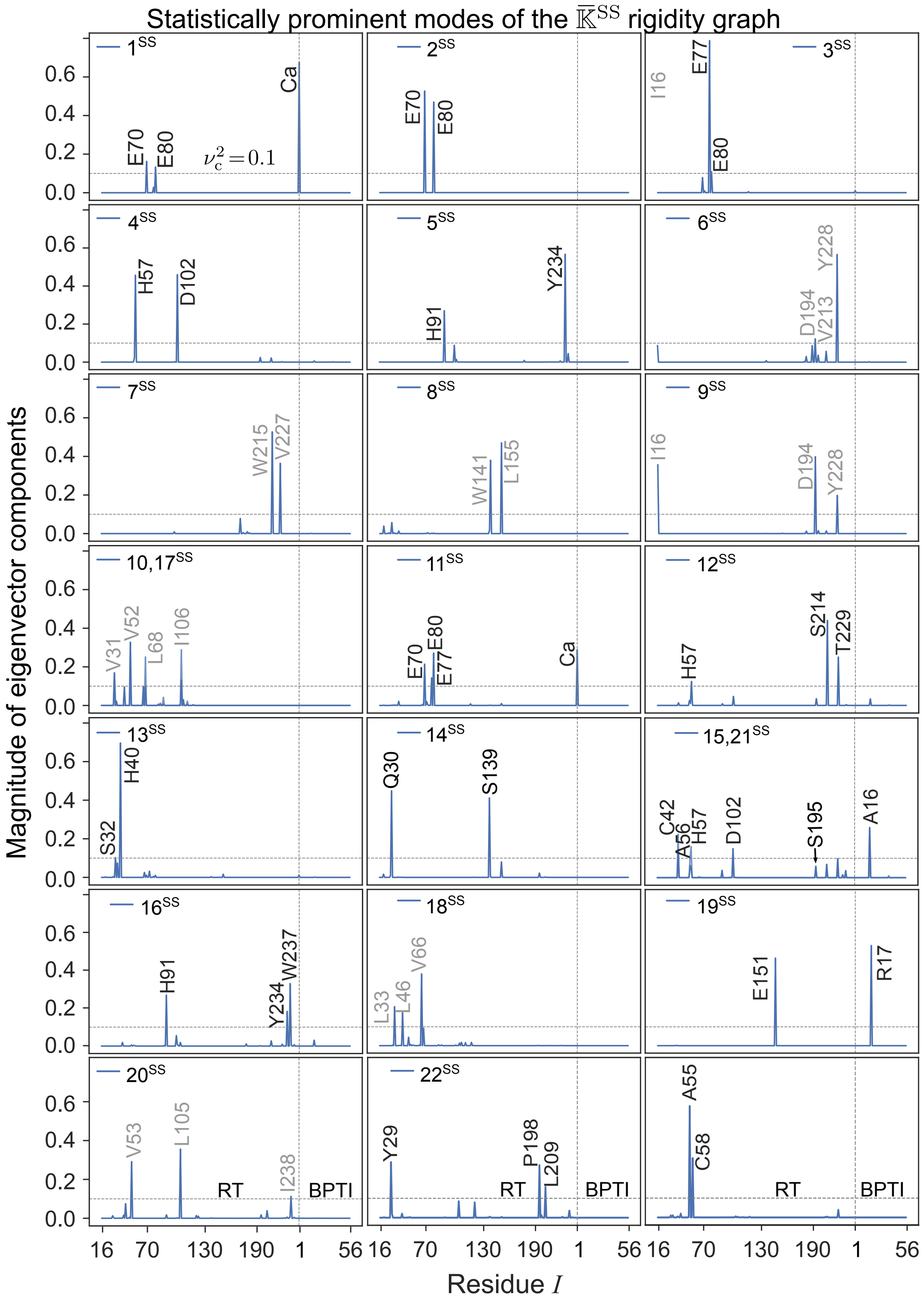

Figure S6: Prominent modes of the RT $\bar{\mathbb{K}}^{\mathrm{SS}}$ rigidity graph. The magnitude of the eigenvector component for each residue is shown. The dotted horizontal line is the magnitude cutoff for identifying the significantly contributing residues of each eigenvector. The residues labelled in grey are hydrophobic interactions. The dotted vertical line separates the eigenvector components for RT and BPTI residues. The residues are indexed based on the numbering in PDB ID: 3TGI.

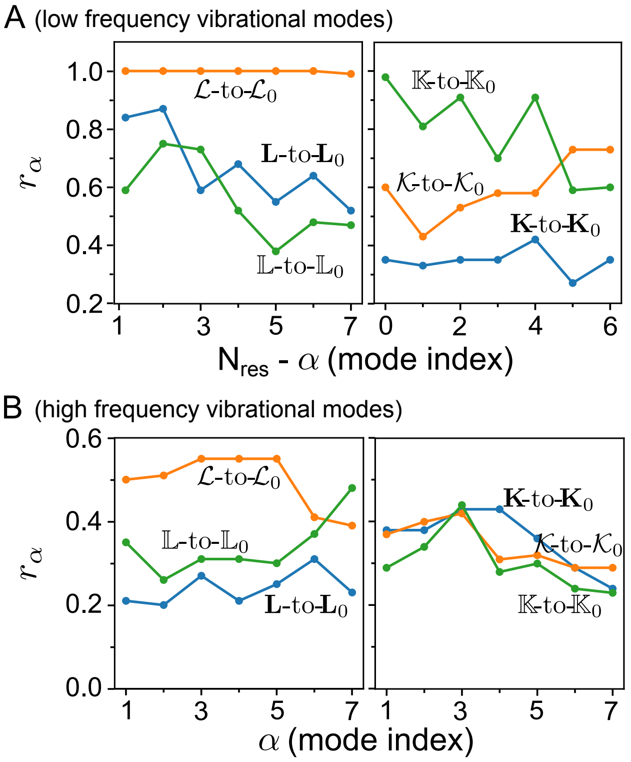

Figure S7: The graph similarity(r_α) between bsENM$_0$ and bsENM for rigidity graph and laplacian matrix of full, skeleton, and non-skeleton springs. (A) Left panel: the r_α between bsENM$_0$ and bsENM of the 7 lowest frequency modes of full ($\mathbf{L}$-to-$\mathbf{L}_0$), skeleton ($\mathcal{L}$-to-$\mathcal{L}_0$), non-skeleton ($\mathbb{L}$-to-$\mathbb{L}_0$) springs of the Laplacian matrix. Right panel: the r_α between bsENM$_0$ and bsENM of the 7 lowest frequency modes of full ($\mathbf{K}$-to-$\mathbf{K}_0$), skeleton ($\mathcal{K}$-to-$\mathcal{K}_0$), non-skeleton ($\mathbb{K}$-to-$\mathbb{L}_0$) springs of the signless Laplacian matrix. The first time window is used for this analysis. (B) Left panel: the r_α between bsENM$_0$ and bsENM of the 7 highest frequency modes of full ($\mathbf{L}$-to-$\mathbf{L}_0$), skeleton ($\mathcal{L}$-to-$\mathcal{L}_0$), non-skeleton ($\mathbb{L}$-to-$\mathbb{L}_0$) springs of the Laplacian matrix. Right panel: the r_α between bsENM$_0$ and bsENM of the 7 highest frequency modes of full ($\mathbf{K}$-to-$\mathbf{K}_0$), skeleton ($\mathcal{K}$-to-$\mathcal{K}_0$), non-skeleton ($\mathbb{K}$-to-$\mathbb{L}_0$) springs of the signless Laplacian matrix. The first time window is used for this analysis.

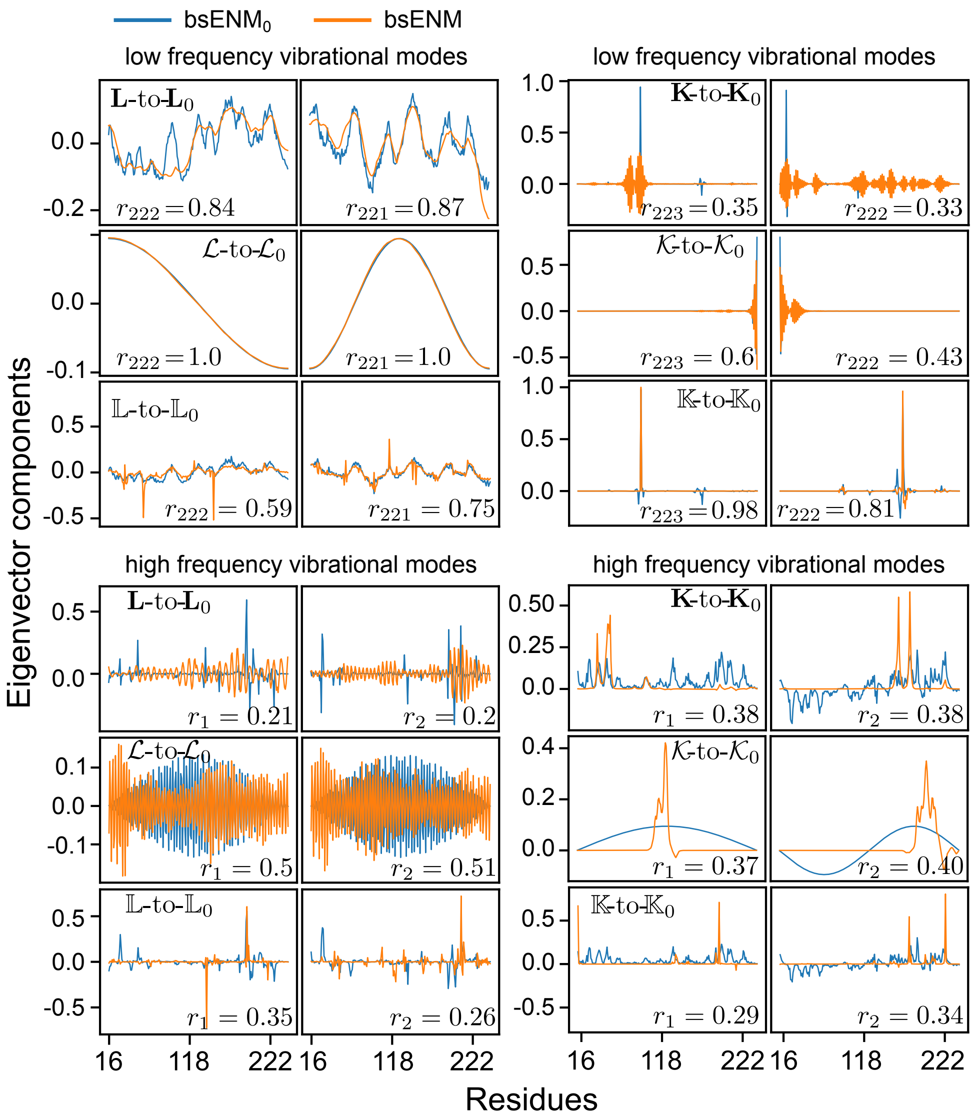

Figure S8: Comparison of mode shapes between bsENM$_0$ and bsENM for rigidity graph and laplacian matrix of full, skeleton, and non-skeleton springs. Top left: the mode shapes of bsENM$_0$ and bsENM for full (**L**-to-**L**$_0$), skeleton ($\mathcal{L}$-to-$\mathcal{L}_0$) and non-skeleton (𝕃-to-𝕃$_0$) of the 2 lowest frequency modes in the rigidity graph. Top right: the mode shapes of bsENM$_0$ and bsENM for full (**K**-to-**K**$_0$), skeleton ($\mathcal{K}$-to-$\mathcal{K}_0$) and non-skeleton (𝕂-to-𝕂$_0$) of the 2 lowest frequency modes in the rigidity graph. Bottom left: the mode shapes of bsENM$_0$ and bsENM for full (**L**-to-**L**$_0$), skeleton ($\mathcal{L}$-to-$\mathcal{L}_0$) and non-skeleton (𝕃-to-𝕃$_0$) of the 2 highest frequency modes in the rigidity graph. Bottom right: the mode shapes of bsENM$_0$ and bsENM for full (**K**-to-**K**$_0$), skeleton ($\mathcal{K}$-to-$\mathcal{K}_0$) and non-skeleton (𝕂-to-𝕂$_0$) of the 2 highest frequency modes in the rigidity graph. The first time window is used for this analysis.

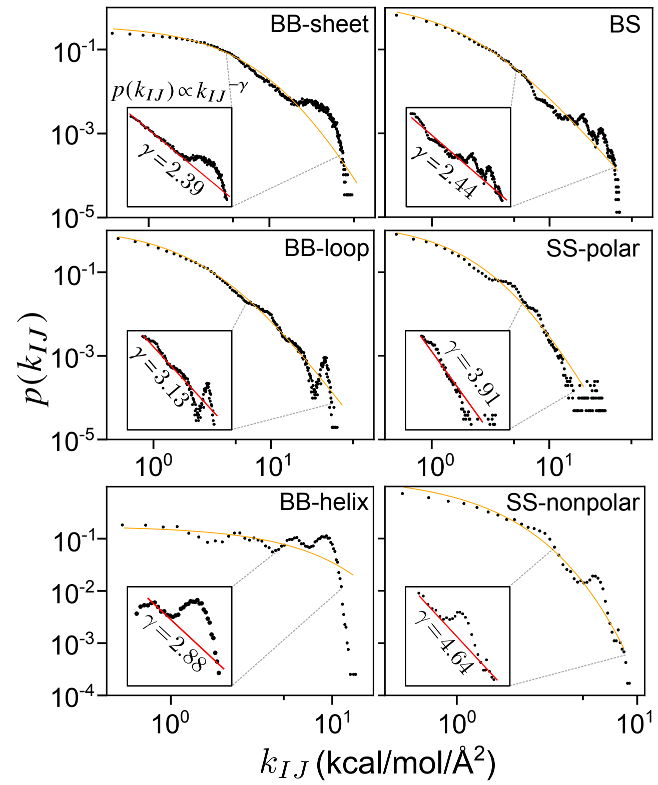

Figure S9: Distribution of k_{IJ} in RT for different categories based on the type of inter-residue interactions. The off-diagonal (k_{IJ}) terms of $\mathbb{K}^{BB}$ are grouped according to the secondary structure (BB-sheet, BB-helix, and BB-loop) while those of $\mathbb{K}^{SS}$ are divided into SS-polar and SS-nonpolar groups. The $\mathbb{K}^{BS}$ couplings are all polar since backbone is involved. If either residue I or J is not in a helix or sheet, the pair is counted as in loop.

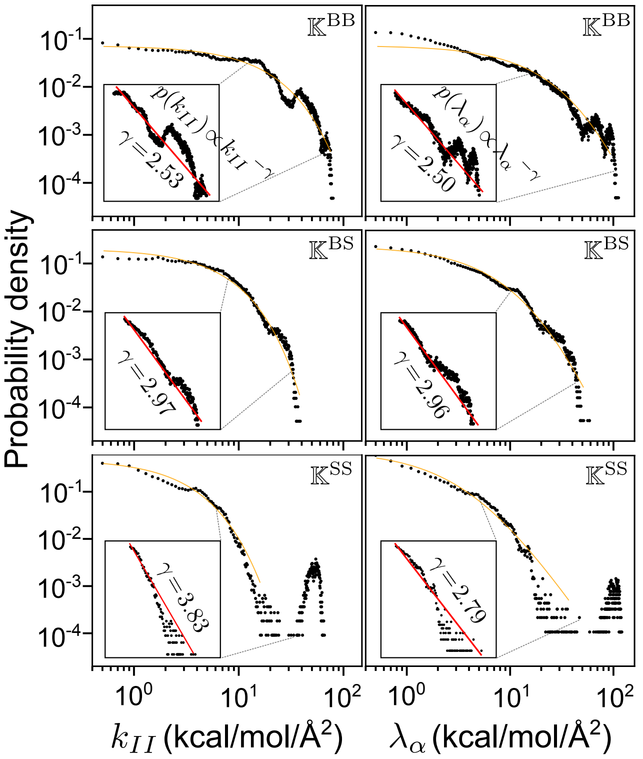

Figure S10: The $p(k_{II})$ (left panels) and $p(\lambda_\alpha)$ (right panels) of $\mathbb{K}^{BB}$, $\mathbb{K}^{BS}$, and $\mathbb{K}^{SS}$ rigidity graphs on a log-log scale. The rigidity graphs of all trajectory windows in the 5 μs production run of PDZ3 are used. The orange line is the best-fit Lomax distribution for the heavy-tailed profiles. The red line in the insert is a power-law fit with the scaling exponent γ.

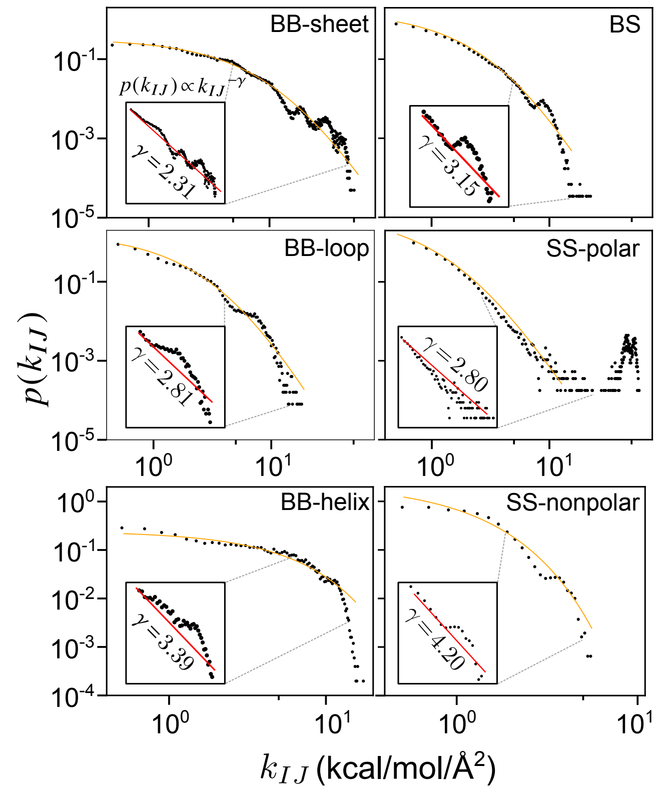

Figure S11: Distribution of k_{IJ} in PDZ3 for different categories based on the type of inter-residue interactions. The off-diagonal (k_{IJ}) terms of $\mathbb{K}^{\mathrm{BB}}$ are grouped according to the secondary structure (BB-sheet, BB-helix, and BB-loop) while those of $\mathbb{K}^{\mathrm{SS}}$ are divided into SS-polar and SS-nonpolar groups. The $\mathbb{K}^{\mathrm{BS}}$ couplings are all polar since backbone is involved. If either residue I or J is not in a helix or sheet, the pair is counted as in loop.

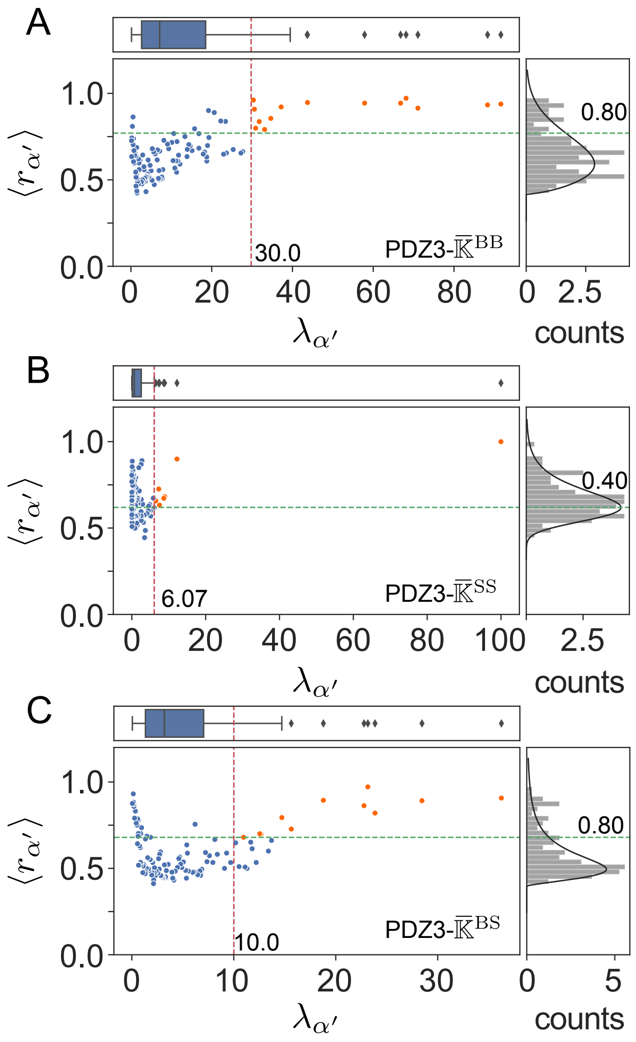

Figure S12: For PDZ3, identification of the statistically prominent modes in a $\overline{\mathbb{K}}$ rigidity graph during protein dynamics from the distribution of eigenvalue ($\lambda_{\alpha'}$) and averaged mean-mode content ($\langle r_{\alpha'} \rangle$). (A) $\overline{\mathbb{K}}^{BB}$, (B) $\overline{\mathbb{K}}^{SS}$, (C) $\overline{\mathbb{K}}^{BS}$. The box plot on the top shows the distribution of $\lambda_{\alpha'}$ and that on the right shows the distribution of $\langle r_{\alpha'} \rangle$. The red dotted lines indicate the cutoff of $\lambda_{\alpha'}$ for an exceptionally strong mode. The green dotted lines indicate the cumulative density cutoff used for identifying modes with high $\langle r_{\alpha'} \rangle$ from the distribution fit. The orange markers indicate the identified prominent modes.

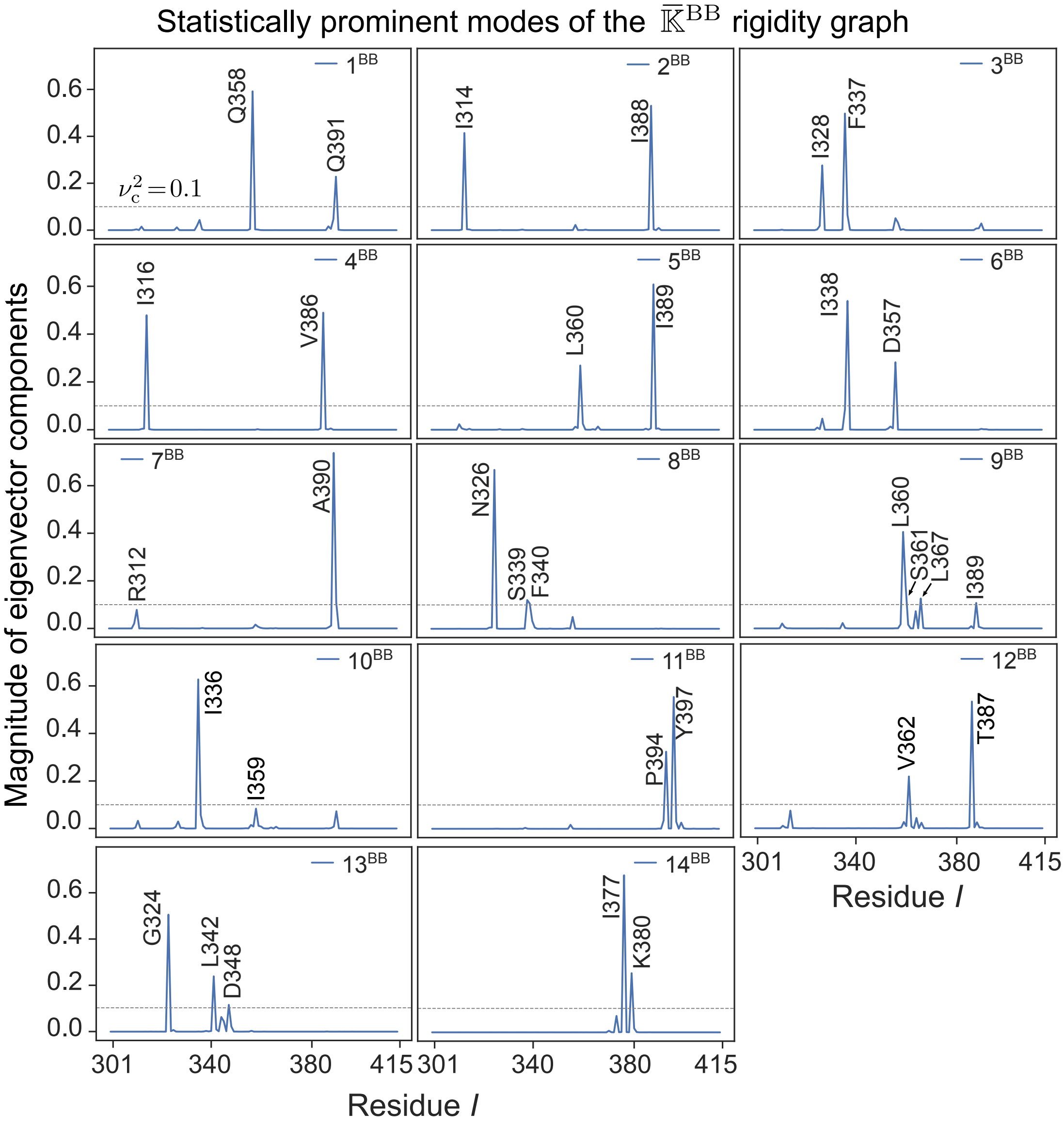

Figure S13: Prominent modes of the PDZ3 $\overline{\mathbb{K}}^{\mathrm{BB}}$ rigidity graph. The magnitude of the eigenvector component for each residue is shown. The dotted horizontal line is the magnitude cutoff for identifying the significantly contributing residues of each eigenvector. The residues are indexed based on the numbering in PDB ID: 1BEF.

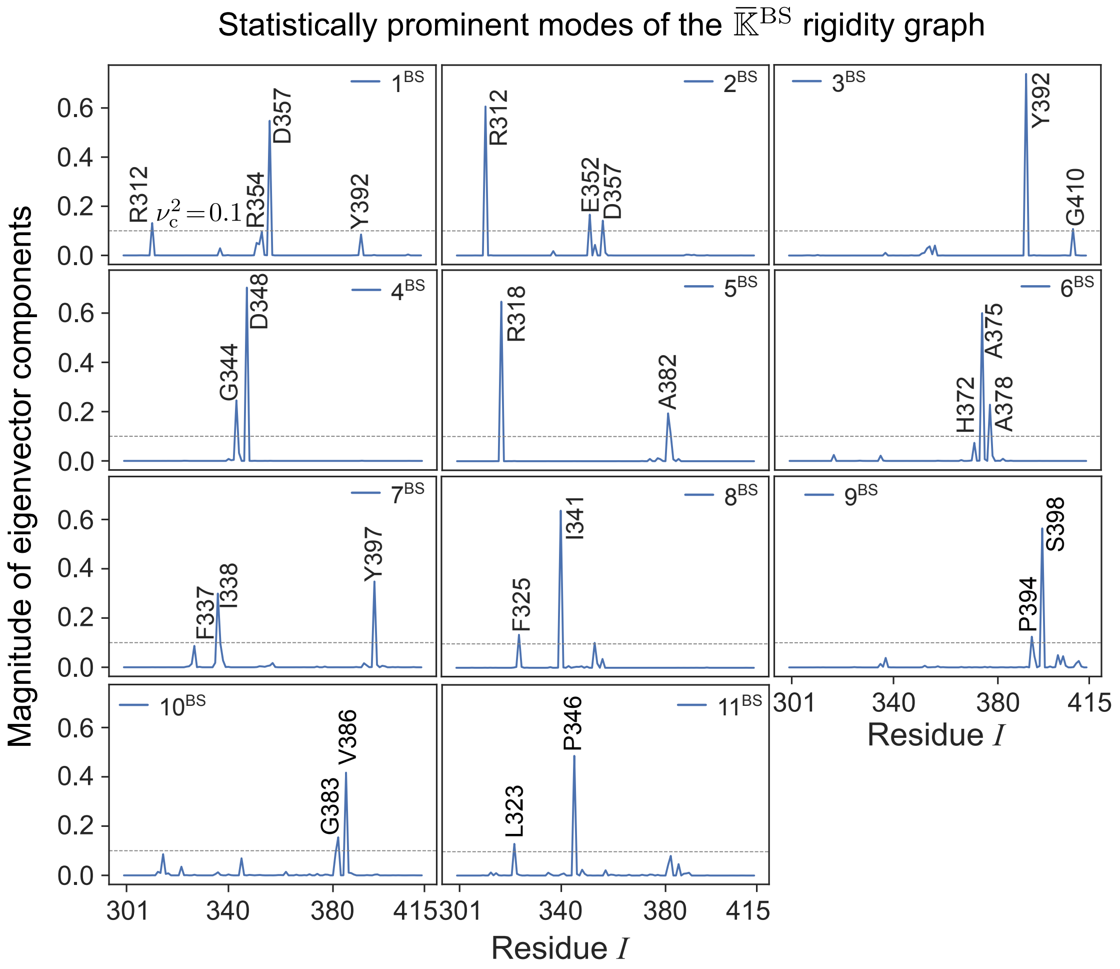

Figure S14: Prominent modes of the PDZ3 $\overline{\mathbb{K}}^{\mathrm{BS}}$ rigidity graph. The magnitude of the eigenvector component for each residue is shown. The dotted horizontal line is the magnitude cutoff for identifying the significantly contributing residues of each eigenvector. The residues are indexed based on the numbering in PDB ID: 1BEF.

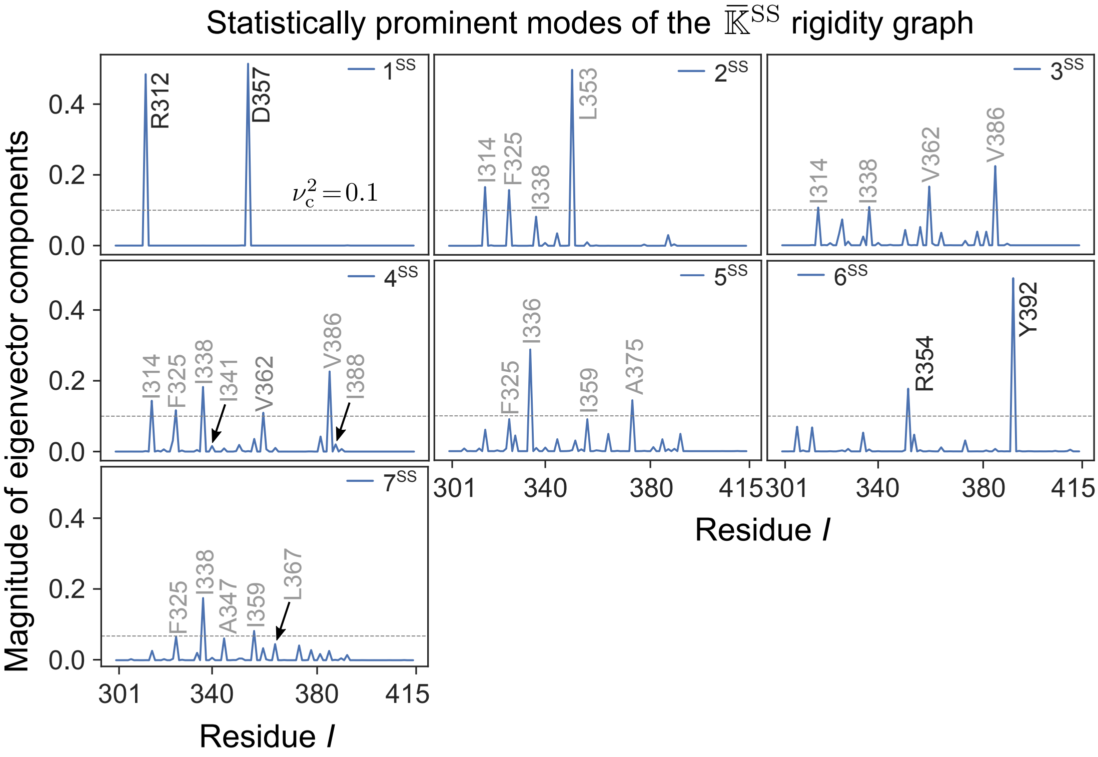

Figure S15: Prominent modes of the PDZ3 $\overline{\mathbb{K}}^{\mathrm{SS}}$ rigidity graph. The magnitude of the eigenvector component for each residue is shown. The dotted horizontal line is the magnitude cutoff for identifying the significantly contributing residues of each eigenvector. The residues are indexed based on the numbering in PDB ID: 1BEF.

A

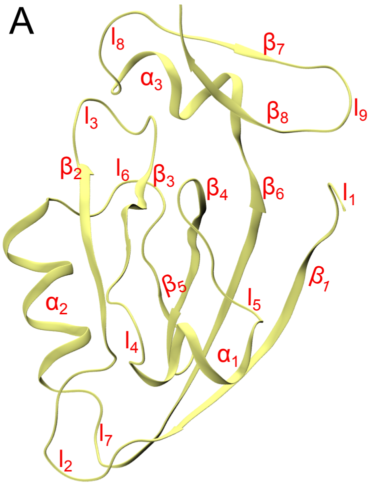

Secondary structure	Range	Secondary structure	Range
β_1	R312 − R318	α_3	P394 − R399
β_2	F325 − G329	l_1	F301 − P311
β_3	I336 − I341	l_2	G319 − G324
β_4	D357 − V362	l_3	G330 − G335
β_5	V365 − L367	l_4	L342 − G345
β_6	T385 − Y392	l_5	G351 − G356
β_7	S404 − V406	l_6	R368 − S371
β_8	I412 − N415	l_7	N381 − Q384
α_1	P346 − S350	l_8	F400 − N403
α_2	H372 − K380	l_9	N407 − R411

B

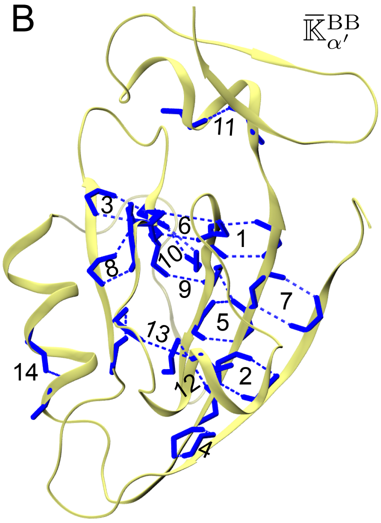

α'	$\lambda_{\alpha'}$	Residues
3^{BB}	71.0	I328-F337
6^{BB}	57.8	I338-**D357**
10^{BB}	33.1	**I336**-I359
1^{BB}	91.6	Q358-Q391
5^{BB}	66.7	L360-I389
9^{BB}	34.6	L360-S361-**L367**-I389
12^{BB}	30.8	**V362**-T387
8^{BB}	37.2	N326-S339-F340
13^{BB}	30.5	G324-L342-D348
14^{BB}	30.3	I377-**K380**
2^{BB}	88.3	I314-I388
4^{BB}	68.1	I316-V386
7^{BB}	43.7	**R312**-A390
11^{BB}	31.7	**P394**-Y397
	(kcal/mol/Å^2)	

Figure S16: Prominent modes of the PDZ3 $\overline{\mathbb{K}}^{\mathrm{BB}}$ rigidity graph. (A) The protein structure and the residue composition of secondary structures. (B) Prominent mode residues are in licorice. The indices, eigenvalue, and residues of prominent modes are listed.

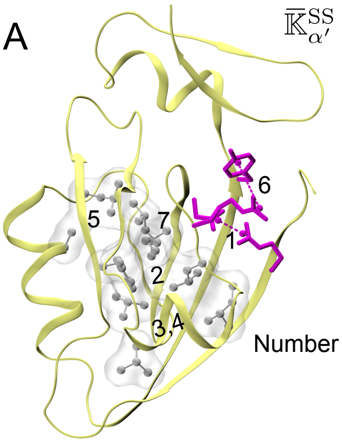

α'	$\lambda_{\alpha'}$	Residues
2^{SS}	12.2	**F325**-I338-L353-I314
3^{SS}	8.9	I314-I338-**V362**-V386
4^{SS}	8.6	**F325**-**I341**-I338-**V362**-V386-I388
7^{SS}	6.4	**F325**-I338-A347-I359-**L367**
5^{SS}	7.4	**F325**-**I336**-I359-A375
1^{SS}	99.9	**R312**-**D357**
6^{SS}	7.2	R354-**Y392**
	(kcal/mol/Å^2)	

Number of PDZ3's prominent mode residues in		BB	SS	BS
	β/α interior	21	8	7
	β/α periphery	7	8	9
	loop	2	2	7

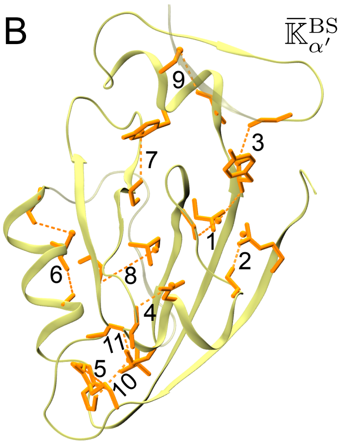

α'	$\lambda_{\alpha'}$	Residues
6^{BS}	18.7	**H372**-A375-A378
5^{BS}	22.7	**R318**-A382
10^{BS}	12.5	G383-V386
11^{BS}	10.9	L323-P346
4^{BS}	23.1	G344-D348
8^{BS}	14.7	**F325**-**I341**
1^{BS}	36.2	**R312**-R354-**D357**-**Y392**
2^{BS}	28.4	**R312**-E352
3^{BS}	23.8	**Y392**-G410
7^{BS}	15.6	F337-Y397
9^{BS}	12.7	P394-S398
	(kcal/mol/Å^2)	

Figure S17: Prominent modes in (A) $\bar{\mathbb{K}}^{SS}$ and (B) $\bar{\mathbb{K}}^{BS}$ rigidity graphs of PDZ3. The PDZ3 residues in hydrogen bonds and salt bridges are in licorice and those in hydrophobic couplings are in ball-and-stick. The indices, eigenvalues, and residues of prominent modes are listed. Residues in prominent modes are hotspots in the mechanical coupling network, and the numbers within a secondary structure, β/α interior, at a secondary structure periphery, β/α periphery, or in a loop are reported.

A

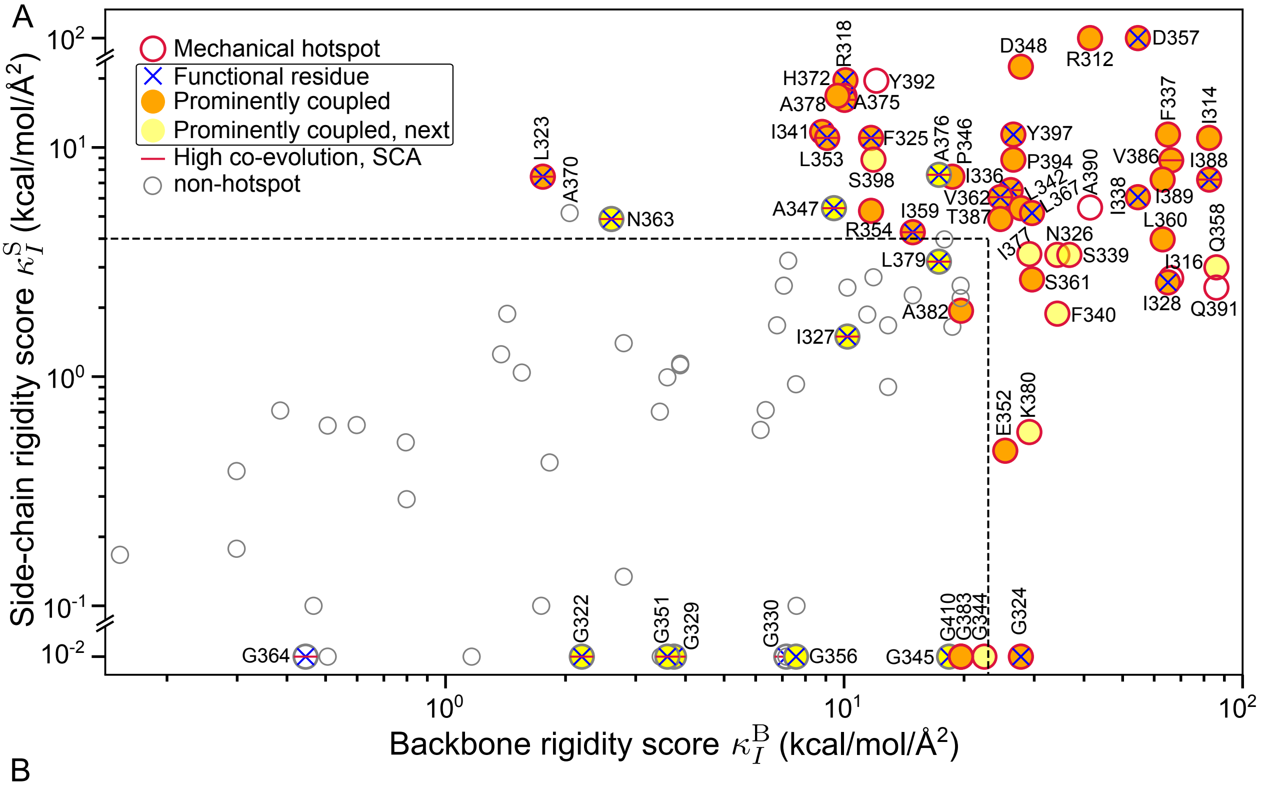

B

Experimentally established functional residues		
Residues	Function established in experiments	References
R318,L323,G324,F325,I327,I328, G329,G330,I336,I338,I341,A347, L353,I359,V362,L367,H372,A375, A376,L379,I388	Mutation sensitive	7,8
G322,L323,G324,F325,I327,G329, I338,I341,G345,A347,G351,L353, G356,D357,I359,V362,N363,G364, H372,A375,A376,L379,I388	Ultra conservation	9
G322,L323,F325,I327,G329,G330, I336,A347,G351,L353,I359,V362, N363,G364,H372,A375,A376,L379, V386,I388	High co-evolution, SCA	9

Figure S18: For PDZ3, (A) the rigidity score during protein dynamics in backbone (κ_I^B) and in side chain (κ_I^S) for residues. Red circles are mechanical hotspots. Dashed lines are the low-end scores coming from $\overline{\mathbb{K}}^{BB}$ and $\overline{\mathbb{K}}^{SS}$ prominent modes. Labelled × as "functional residue" are those listed in (B) that have experimentally verified function[7, 8] or ultra high conservation in MSA[9]; residues in both are underlined. Filled orange as "prominently coupled" are the mechanical hotspots of a single rigidity graph mode that have least one functional residue. Filled yellow as "prominently coupled, next" are the hotspots next to a functional residue in sequence or non-hotspot functional residues next to a hotspot. Labeled — are residues having high co-evolution in SCA, cf. (B). Grey circles are non-hotspot residues.

Table S1: The atomic-to-CG mapping for the bsENM. The CG sites in bsENM serve to read out the inter-site statistics of elastic couplings from the all-atom MD trajectory. The assignment of CG sites thus do not affect the quality of all-atom protein dynamics. The goal is to capture significant interaction patterns that can survive thermal noise, and the CG sites are thus located where specific molecular interactions are typically observed in the all-atom MD trajectories. As an example, the CG site of threonine side chain is placed on O_{γ_1}, but the hydrophobic interactions of the side chain are fully retained in the all-atom MD trajectory and can still be detected by the CG site if the atomic fluctuations are due to such interactions. Inspection of the all-atom MD trajectories indicates that the specific interactions of threonine are mostly due to the hydroxyl group but force constants can still be observed with hydrophobic residues. For tyrosine and tryptophan side chains, the center of mass CG sites also allow prominent modes due to hydrogen bonding as well as hydrophobic coupling to be captured. In the protein systems studied here, methionine residues are generally solvent exposed and the atomic fluctuations gave rise to low spring constant values.

**backbone CG sites**	N & O
**side-chain CG site**<br>(Center of mass if more than one atoms)	
Lysine	N_ζ
Aspartic acid	O_{δ_1} O_{δ_2}
Glutamic acid	O_{ϵ_1} O_{ϵ_2}
Glutamine	O_{ϵ_1} N_{ϵ_2}
Asparagine	O_{δ_1} N_{δ_2}
Histidine	C_γ N_{δ_1} C_{δ_2} C_{ϵ_1} N_{ϵ_2}
Serine	O_γ
Threonine	O_{γ_1}
Tyrosine	C_γ C_{δ_1} C_{δ_2} C_{ϵ_1} C_{ϵ_2} C_{ζ_2} O_η
Cysteine	S_γ
Tryptophan	C_γ C_{δ_1} C_{δ_2} N_ϵ C_{ϵ_2} C_{ϵ_3} C_{ζ_2} C_{ζ_3} C_{η_2}
Alanine	C_β
Isoleucine	C_{γ_1} C_{γ_2} C_δ
Leucine	C_{δ_1} C_{δ_2}
Methionine	S_δ
Phenylalanine	C_γ C_{δ_1} C_{δ_2} C_{ϵ_1} C_{ϵ_2} C_ζ
Valine	C_{γ_1} C_{γ_2}
Proline	C_γ
Arginine	N_{η_1} N_{η_2}
Glycine	-

Table S2: The coupling strength (k_{ij}) of disulfide bonds.The $S_\gamma - S_\gamma$ spring constant in the charmm all-atom forcefield is 173.0 kcal/mol/$\mathring{A}^2$.

$S_\gamma - S_\gamma$	Time averaged k_{ij}
C168–C182	170.35
C22–C157	169.01
C42–C58	169.09
C191–C220	168.52
C128–C232	168.33
C136–C201	166.81

Table S3: The functional residues of RT. Residues with experimentally verified functional impact are listed with the corresponding references. Functional residues also include those having ultra conservation in MSA. Residues in both cases are underlined.

Residues	Function established in experiment	References
H57,D102,S195	Active site	10–13
I16,G193,D194,S214, C220,P225	Catalytic Activity	14–19
Y172,D189,S190,C191, G216,P225,D226	Substrate Specificity	9, 19–23
I16,D194	Enzyme Activation	24
A55,A56,N143,E151	Substrate binding	25–27
E70,N72,V75,E77,E80	Ca^{2+} binding loop	28, 29
C42,C58,M104,L105, C136,C157,Q210,T229	Thermal Stability	9, 30
H40,W215	Active site access	31, 32
R117,S146	Autolysis control	33–35
I16,P28,Y29,C42,G43,G44, W51,S54,A55,A56,H57,C58, H91,D102,G140,W141,G142, C168,C182,C191,G193,D194, S195,G196,G197,P198,G211, S214,W215,C220,P225,Y228, W237,I238	Ultra conservation	36

Table S4: Residues showing high co-evolution in SCA (statistical coupling analysis) or DCA (direct coupling analysis). The residues categorized as sectors in SCA are colored accordingly.

Residues	Method	References
I16, G19, T21, N25, S26, V27, P28, Y29, Q30, V31, S32, N34, H40, C42, G43, G44, L46, I47, W51, V52, V53, S54, A55, A56, H57, C58, L68, G69, H71, E77, Q81, A85, I89, H91, P92, F94, D95, N101, D102, M104, L105, K107, L108, L114, V118, L123, P124, C136, I138, S139, G140, W141, G142, P152,L155, Q156, A160, P161, C168, Y172, I176, T177, M180, C182, V183, G184, K188A,D189, C191, Q192, G193, D194, S195, G196, G197, P198, V199, V200, C201, G211, V213, S214, W215, G216, C220, A221, P222, P225, G226, V227, Y228,T229, K230, Y234, W237, I238	Statistical coupling analysis (top 6 IC modes, IC1, IC2 and IC3 corresponds to green, red and blue sector respectively as determined by Halabi et al., 2009. ICs 4-6 follow the general principle of physical connectivity that seems to characterize sectors, but their functional meaning is yet to be determined.)	9, 36
S26, V27, Q30, S32, N34, H40, C42, G44, S45, L46, W51, V52, V53, H57, C58, Y59, H71, N72, E77, G78, N79, N100,D102, M104, L105, V112, C136, I138, S139, D157, P161, N179, G184, D189, S190, C191, S195, C201, L209, V213, C220, G226, Y228	Direct coupling analysis (true contact pairs, pairs with distance less than 8.0 Å)	37

References

[1] A. Pasternak, D. Ringe, L. Hedstrom, Comparison of anionic and cationic trypsinogens: The anionic activation domain is more flexible in solution and differs in its mode of BPTI binding in the crystal structure, Protein Sci. 8 (1) (1999) 253–258. doi:0.1110/ps.8.1.253.

[2] D. A. Doyle, A. Lee, J. Lewis, E. Kim, M. Sheng, R. MacKinnon, Crystal structures of a complexed and peptide-free membrane protein-binding domain: molecular basis of peptide recognition by PDZ, Cell 85 (7) (1996) 1067–76. doi:10.1016/s0092-8674(00)81307-0.

[3] R. B. Best, X. Zhu, J. Shim, P. E. M. Lopes, J. Mittal, M. Feig, J. MacKerell, Alexander D., Optimization of the additive CHARMM all-atom protein force field targeting improved sampling of the backbone phi, psi and side-chain chi1 and chi2 dihedral angles, J. Chem. Theory Comput. 8 (9) (2012) 3257–3273. doi:10.1021/ct300400x.

[4] M. J. Abraham, T. Murtola, R. Schulz, S. Páll, J. C. Smith, B. Hess, E. Lindahl, GROMACS: High performance molecular simulations through multi-level parallelism from laptops to supercomputers, SoftwareX 1-2 (2015) 19 – 25. doi:https://doi.org/10.1016/j.softx.2015.06.001.

[5] B. Hess, H. Bekker, H. J. C. Berendsen, J. G. E. M. Fraaije, Lincs: A linear constraint solver for molecular simulations, J. Comput. Chem. 18 (12) (1997) 1463–1472. doi:10.1002/(SICI)1096-987X(199709)18:12<1463::AID-JCC4>3.0.CO;2-H.

[6] B. R. Brooks, D. Janežič, M. Karplus, Harmonic Analysis of Large Systems. I. Methodology, J. Comput. Chem. 16 (12) (1995) 1522–1542. doi:10.1002/jcc.540161209.

[7] C. N. Chi, A. Engström, S. Gianni, M. Larsson, P. Jemth, Two conserved residues govern the salt and ph dependencies of the binding reaction of a pdz domain, J Biol Chem 281 (48) (2006) 36811–8. doi:10.1074/jbc.M607883200.

[8] J. McLaughlin, Richard N., F. J. Poelwijk, A. Raman, W. S. Gosal, R. Ranganathan, The spatial architecture of protein function and adaptation, Nature 491 (7422) (2012) 138–U163. doi:10.1038/nature11500.

[9] N. Halabi, O. Rivoire, S. Leibler, R. Ranganathan, Protein sectors: evolutionary units of three-dimensional structure., Cell 138 (4) (2009) 774–786. doi:10.1016/j.cell.2009.07.038.

[10] L. A. Pelc, Z. Chen, D. W. Gohara, A. D. Vogt, N. Pozzi, E. Di Cera, Why ser and not thr brokers catalysis in the trypsin fold, Biochemistry 54 (7) (2015) 1457–1464. doi:10.1021/acs.biochem.5b00014.

[11] T. Earnest, E. Fauman, C. S. Craik, R. Stroud, 1.59 a structure of trypsin at 120 k: comparison of low temperature and room temperature structures, Proteins 10 (3) (1991) 171–87. doi:10.1002/prot.340100303.

[12] C. Heinis, A. Huber, S. Demartis, J. Bertschinger, S. Melkko, L. Lozzi, P. Neri, D. Neri, Selection of catalytically active biotin ligase and trypsin mutants by phage display, Protein Eng. 14 (12) (2001) 1043–1052. doi:10.1093/protein/14.12.1043.

[13] W. Y. Wahlgren, G. Pal, J. Kardos, P. Porrogi, B. Szenthe, A. Patthy, L. Graf, G. Katona, The catalytic aspartate is protonated in the michaelis complex formed between trypsin and an in vitro evolved substrate-like inhibitor a refined mechanism of serine protease action, J. Biol. Chem. 286 (5) (2011) 3587–3596. doi:10.1074/jbc.M110.161604.

[14] D. R. Corey, M. E. Mcgrath, J. R. Vasquez, R. J. Fletterick, R J, C. S. Craik, An alternate geometry ffor the catalytic triad of serine proteases, J. Am. Chem. Soc. 114 (12) (1992) 4905–4907. doi:10.1021/ja00038a067.

[15] M. M. Krem, S. Prasad, E. Di Cera, Ser(214) is crucial for substrate binding to serine proteases, J. Biol. Chem. 277 (43) (2002) 40260–40264. doi:10.1074/jbc.M206173200.

[16] M. E. McGrath, J. R. Vásquez, C. S. Craik, A. S. Yang, B. Honig, R. J. Fletterick, Perturbing the polar environment of asp102 in trypsin: consequences of replacing conserved ser214, Biochemistry 31 (12) (1992) 3059–64. doi:10.1021/bi00127a005.

[17] A. E. Schmidt, M.-f. Sun, T. Ogawa, S. P. Bajaj, D. Gailani, Functional role of residue 193 (chymotrypsin numbering) in serine proteases: Influence of side chain length and beta-branching on the catalytic activity of blood coagulation factor xia, Biochemistry 47 (5) (2008) 1326–1335. doi:10.1021/bi701594j.

[18] V. Varallyay, Z. Lengyel, L. Graf, L. Szilagyi, The role of disulfide bond c191-c220 in trypsin and chymotrypsin, Biochem. Biophys. Res. Commun. 230 (3) (1997) 592–596. doi:10.1006/bbrc.1996.6009.

[19] E. R. Guinto, S. Caccia, T. Rose, K. Futterer, G. Waksman, E. Di Cera, Unexpected crucial role of residue 225 in serine proteases, Proc. Natl. Acad. Sci. U.S.A. 96 (5) (1999) 1852–1857. doi:10.1073/pnas.96.5.1852.

[20] J. J. Perona, L. Hedstrom, W. J. Rutter, R. J. Fletterick, Structural origins of substrate discrimination in trypsin and chymotrypsin, Biochemistry 34 (5) (1995) 1489–99. doi:10.1021/bi00005a004.

[21] B. Jelinek, J. Antal, I. Venekei, L. Gráf, Ala226 to gly and ser189 to asp mutations convert rat chymotrypsin b to a trypsin-like protease, Protein Eng Des Sel 17 (2) (2004) 127–31. doi:10.1093/protein/gzh014.

[22] L. B. Evnin, J. R. Vásquez, C. S. Craik, Substrate specificity of trypsin investigated by using a genetic selection, Proc. Natl. Acad. Sci. U.S.A. 87 (17) (1990) 6659–63. doi:10.1073/pnas.87.17.6659.

[23] L. Hedstrom, J. J. Perona, W. J. Rutter, Converting trypsin to chymotrypsin: residue 172 is a substrate specificity determinant., Biochemistry 33 (29) (1994) 8757–8763. doi:10.1021/bi00195a017.

[24] B. M. Stojanovski, Z. Chen, S. K. Koester, L. A. Pelc, E. Di Cera, Role of the I16-D194 ionic interaction in the trypsin fold, Sci. Rep. 9 (18035) (December 2019). doi:10.1038/s41598-019-54564-6.

[25] W. S. Willett, L. S. Brinen, R. J. Fletterick, C. S. Craik, Delocalizing trypsin specificity with metal activation, Biochemistry 35 (19) (1996) 5992–5998. doi:10.1021/bi9530191.

[26] M. Takeda-Shitaka, H. Umeyama, Elucidation of the cause for reduced activity of abnormal human plasmin containing an ala55-thr mutation: importance of highly conserved ala55 in serine proteases., FEBS Lett 425 (3) (1998) 448–452. doi:10.1016/s0014-5793(98)00280-4.

[27] F. C. Peterson, N. C. Gordon, P. G. Gettins, High-level bacterial expression and 15n-alanine-labeling of bovine trypsin. application to the study of trypsin-inhibitor complexes and trypsinogen activation by nmr spectroscopy, Biochemistry 40 (21) (2001) 6275–83. doi:10.1021/bi0100992.

[28] P. Goettig, H. Brandstetter, V. Magdolen, Surface loops of trypsin-like serine proteases as determinants of function, Biochimie 166 (2019) 52–76. doi:10.1016/j.biochi.2019.09.004.

[29] T. Sipos, J. R. Merkel, An effect of calcium ions on the activity, heat stability, and structure of trypsin, Biochemistry 9 (14) (1970) 2766–75. doi:10.1021/bi00816a003.

[30] T. T. Baird, Jr, W. D. Wright, C. S. Craik, Conversion of trypsin to a functional threonine protease, Protein Sci 15 (6) (2006) 1229–38. doi:10.1110/ps.062179006.

[31] T. Kromann-Hansen, E. L. Lange, H. P. Sørensen, G. Hassanzadeh-Ghassabeh, M. Huang, J. K. Jensen, S. Muyldermans, P. J. Declerck, E. A. Komives, P. A. Andreasen, Discovery of a novel conformational equilibrium in urokinase-type plasminogen activator, Sci. Rep. 7 (1) (2017) 1–11. doi:10.1038/s41598-017-03457-7.

[32] P. S. Gandhi, M. J. Page, Z. Chen, L. Bush-Pelc, E. Di Cera, Mechanism of the anticoagulant activity of thrombin mutant w215a/e217a, J Biol Chem 284 (36) (2009) 24098–105. doi:10.1074/jbc.M109.025403.

[33] E. Várallyay, G. Pál, A. Patthy, L. Szilágyi, L. Gráf, Two mutations in rat trypsin confer resistance against autolysis, Biochem. Biophys. Res. Commun. 243 (1) (1998) 56–60. doi:10.1006/bbrc.1997.8058.

[34] X. F. Li, X. Nie, J. G. Tang, Anti-autolysis of trypsin by modification of autolytic site Arg117, Biochem. Biophys. Res. Commun. 250 (2) (1998) 235–9. doi:10.1006/bbrc.1998.9295.

[35] A. Bódi, G. Kaslik, I. Venekei, L. Gráf, Structural determinants of the half-life and cleavage site preference in the autolytic inactivation of chymotrypsin, Eur. J. Biochem. 268 (23) (2001) 6238–46. doi:10.1046/j.0014-2956.2001.02578.x.

[36] O. Rivoire, K. A. Reynolds, R. Ranganathan, Evolution-based functional decomposition of proteins, PLoS Comput. Biol. 12 (6) (2016) e1004817. doi:10.1371/journal.pcbi.1004817.

[37] F. Morcos, A. Pagnani, B. Lunt, A. Bertolino, D. S. Marks, C. Sander, R. Zecchina, J. N. Onuchic, T. Hwa, M. Weigt, Direct-coupling analysis of residue coevolution captures native contacts across many protein families, Proc. Natl. Acad. Sci. U.S.A. 108 (49) (2011) E1293–E1301. doi:10.1073/pnas.1111471108.
